# A Phase 1B Clinical Study of Combretastatin A1 Diphosphate (OXi4503) and Cytarabine (ARA-C) in Combination (OXA) for Patients with Relapsed or Refractory Acute Myeloid Leukemia

**DOI:** 10.3390/cancers12010074

**Published:** 2019-12-26

**Authors:** Fatih M. Uckun, Christopher R. Cogle, Tara L. Lin, Sanjive Qazi, Vuong N. Trieu, Gary Schiller, Justin M. Watts

**Affiliations:** 1 Immuno-Oncology Program, Mateon Therapeutics, Agoura Hills, CA 91301, USA; 2 Ares Pharmaceuticals, St. Paul, MN 55110, USA; 3 Division of Hematology and Oncology, Department of Medicine, College of Medicine & University of Florida Health Cancer Center, University of Florida, Gainesville, FL 32610, USA; 4 Division of Hematologic Malignancies and Cellular Therapeutics, Department of Internal Medicine, University of Kansas Medical Center, University of Kansas Cancer Center and Medical Pavillon, Westwood, KS 66205, USA; 5 Bioinformatics Program and Department of Biology, Gustavus Adolphus College, St Peter, MN 56082, USA; 6 Bone Marrow/Stem Cell Transplantation, Department of Medicine, David Geffen School of Medicine at UCLA, Los Angeles, CA 90095, USA; 7 Department of Medicine, Division of Hematology/Oncology Miller School of Medicine, University of Miami Sylvester Comprehensive Cancer Center, Miami, FL 33136, USA

**Keywords:** AML, combretastatin, leukemia, clinical study, OXA

## Abstract

Combretastatin A1 (OXi4503) is a dual-function drug with vascular disrupting and cytotoxic properties that has exhibited single-agent anti-leukemia activity in murine xenograft models of acute myeloid leukemia (AML) and in a prior Phase 1A clinical study for relapsed/refractory (R/R) AML. The purpose of the present multicenter Phase 1B study was to define the maximum tolerated dose (MTD) and safety profile of OXi4503 and cytarabine (ARA-C) administered in combination (OXA). At four centers, 29 patients with R/R AML or myelodysplastic syndrome (MDS) were treated with OXA. The most common grade 3/4 treatment-emergent adverse events (AEs) were febrile neutropenia (28%), hypertension (17%), thrombocytopenia (17%), and anemia (14%). There were no treatment-emergent grade 5 AEs. Drug-related serious adverse events (SAEs) developed in 4/29 patients (14%) and included febrile neutropenia (*N* = 2), pneumonia/acute respiratory failure (*N* = 1), and hypotension (*N* = 1). 9.76 mg/m^2^ was defined as the MTD of OXi4503 when administered in combination with 1 g/m^2^ ARA-C. In 26 evaluable AML patients, there were 2 complete remissions (CR), 2 complete remissions with incomplete count recovery (CRi) and one partial response (PR), for an overall response rate (ORR) of 19%. The median overall survival (OS) time for the four patients who achieved a CR/CRi was 528 days (95% CI: 434–NA), which was significantly longer than the median OS time of 113 days (95% CI: 77–172) for the remaining 22 patients who did not achieve a CR/CRi (Log Rank Chi Square = 11.8, *p*-value = 0.0006). The safety and early evidence of efficacy of the OXA regimen in R/R AML patients warrant further investigation in a Phase 2 clinical study.

## 1. Introduction

Acute myeloid leukemia (AML) is the most common form of adult acute leukemia with >20,000 estimated new cases and >10,000 deaths in the United States for 2019 (SEER Program, www.seer.cancer.gov). Despite recent advances in therapy, the five-year overall survival remains < 30% and prognosis is grim in patients who experience a recurrence of their disease after first-line induction therapy, with <10% surviving five years after relapse [1,2,3,4,5,6,7,8]. There is an urgent need for effective new treatment strategies for relapsed AML [9,10,11,12,13,14,15,16]. Therefore, clinical development of new targeted medicines, as well as biologics and cellular therapies, has been the focal point of AML research over the last decade, as reflected by several new drug approvals since 2017 [12,13,14,15,16,17,18,19,20,21,22,23,24,25,26,27].

Bone marrow endothelial cells (BMEC) have been implicated by several investigators as protectors of refractory AML clones [28,29,30,31,32,33,34,35]. Bone marrow microvessel density has been implicated as a significant and independent contributor to poor prognosis in AML [31,32]. The endothelial cell-derived proangiogenic protein epithelial growth factor-like 7 (EGFL7) was shown to be involved in an autocrine mechanism, supporting growth and survival of leukemic blasts in patients with AML [33]. Several angiocrine factors have also been implicated in the extrinsic paracrine regulation of AML cells [36,37]. Despite these discoveries, targeting single angiogenic factors in AML has produced minimal to no clinical improvement [38,39,40]. Instead, we hypothesized that a broader anti-vascular strategy may be needed to eliminate the numerous methods of stromal protection in a clinically meaningful manner.

OXi4503 is cis-combretastatin A1 dipotassium diphosphate, a water-soluble prodrug of cis-combretastatin A1 (OXi4500), a naturally occurring derivative of the South African bush willow tree, Combretum caffrum, that reversibly binds tubulin at the colchicine binding site to inhibit microtubule assembly [41,42,43,44,45,46]. OXi4503 is a dual-function drug with vascular disrupting and cytotoxic properties [41,42,43,44,45,46]. The active metabolite of OXi4503 significantly diminishes the chemoprotective effects of BMEC on ARA-C-treated AML cells and exhibits nanomolar cytotoxic activity against human AML cells in vitro [47,48]. OXi4503 exhibited single-agent activity in murine xenograft models of extramedullary leukemia/AML chloroma, as well as in systemic AML models [47,48,49,50].

Notably, the combination of OXi4503 with ARA-C in xenografted human AML models was more effective than either drug alone [47,48]. The clinical safety profile of OXi4503 as a single agent has previously been evaluated in Phase 1A clinical trials [49,50]. In the NCT00977210 Phase 1 dose-finding study in 43 advanced solid tumor patients, OXi4503 doses were escalated from 0.06 to 15.4 mg/m^2^, and 8.5 mg/m^2^ was defined as the MTD [49]. In the NCT01085656 Phase 1 trial designed to evaluate the safety profile, MTD, and recommended Phase 2 dose (RP2D) of OXi4503 in patients with relapsed/refractory (R/R) AML and myelodysplastic syndrome (MDS), a total of 18 patients were treated with single agent OXi4503 and showed a manageable safety profile at single-agent dose levels up to of 7.81 mg/m^2^. There was early evidence of possible single-agent activity as one relapsed AML patient treated at the 2.5 mg/m^2^ dose level achieved a CRi [50].

The primary purpose of the present multicenter Phase 1B study was to define the MTD and safety profile of OXi4503 and ARA-C administered in combination (OXA) in patients with R/R AML.

## 2. Results

### 2.1. Patient Characteristics

We present data from 29 participants with R/R AML or MDS who were treated with OXA between December 2015 and January 2019 (Table S1). The date of data cutoff was 2 June 2019. The baseline patient characteristics are shown in Table 1. 27 patients had R/R AML and 2 had R/R MDS. The median age was 61 years (Range: 26–78 years). Most patients were Caucasian males. 4 patients had relapsed/progressed after 1 prior line of therapy, 12 patients after 2 lines, 4 patients after 3 lines, and 8 patients after 4 or more prior lines (Table 1 and Table S2).

### 2.2. Safety

As of the data cut-off date, safety data were available for all 29 participants who received OXA. All AEs (Table S3), all AEs of CTCAE Grade 3–5 by MedDRA PT (Table S4), all SAEs (Tables S5 and S6) and all CTCAE Grade 3–4 by MedDRA PT laboratory abnormalities (reported as AEs) (Table S7) encountered in all 29 patients treated with OXA regardless of relatedness/causality assessments are shown in the Supplementary Material. The most commonly experienced SAE was febrile neutropenia which was reported in 8 subjects (27.6%) and hypotension in 2 subjects (6.9%). All AEs of any grade related (viz.: definitely, possibly, or probably related) to the study drug, OXi4503, are summarized in Table S8. The most commonly experienced AEs by MedDRA PT related to OXi4503 were febrile neutropenia (27.6%) and hypertension (24.1%), which were medically manageable. The incidence of Grade 3–4 AEs determined to be related to OXi4503 is provided in Table 2 according to dose cohort. Tables S9 and S10 provide a list of all OXi4503-related Grade 3/4 AEs reported along with end of study (EOS) reason, other therapy after EOS, and information on death for the safety population of 29 patients treated with OXA. The related Grade 3–4 AEs were encountered in 17 of the 29 patients (58.6%). The most common Grade 3/4 AEs suspected to be OXi4503-related (occurring in ≥10% of patients) were febrile neutropenia (*N* = 8, 27.6%), hypertension (*N* = 5, 17.2%), decrease in platelet count (*N* = 5, 17.2%), and anemia (*N* = 4, 13.8%) (Table 2). There were no incidences of drug-related Grade 5 AEs. OXi4503-related SAEs were encountered in 4 of the 29 patients (13.8%) and included febrile neutropenia (*N* = 2), pneumonia/acute respiratory failure (*N* = 1), and hypotension (*N* = 1) (Table 3). 9.76 mg/m^2^ was defined as the MTD of OXi4503 when it is used in combination with 1 g/m^2^ ARA-C. At this dose level (Dose cohort #5), 1 subject received 6 doses of OXi4503 (Total cumulative exposure = 59 mg/m^2^), 5 subjects received 2 doses of OXi4503, and 1 subject received 1 dose of OXi4503 (Table S2). Of 7 patients in Dose cohort 5, none developed a treatment-emergent SAE and only 1 patient had a Grade 3 non-hematologic toxicity (viz. hypertension). By comparison, of 4 patients treated in Cohort 6, 2 patients developed SAE and 3 patients had non-hematologic Grade 3–4 AEs, including hypertension (2 patients), hypotension (1 patient), acute hypoxic respiratory failure/pneumonia (1 patient) and liver enzyme elevation (1 patient) (Table 2 and Table 3, Tables S9 and S10). 

### 2.3. Efficacy

The clinical anti-leukemia activity of OXA in evaluable AML patients was also assessed within the confines of a Phase 1 setting. Among the 26 evaluable AML patients, there were 5 patients with an objective response (Age range: 59–77 years, Median = 66 years), including 4 patients who achieved a CR (*N* = 2) or CRi (*N* = 2) and one patient who achieved a PR as their best overall response (Investigator-assessed overall response rate = 19.2%; Table 4). All 4 CR/CRi patients were Caucasian males and 2 had del(5)q along with other adverse risk cytogenetic/molecular abnormalities (Table 4). Three of the 4 CR/CRis were achieved in patients receiving only 1 prior line of therapy, while one patient with CRi (Patient ID: 106–004) had failed 5 previous regimens, including 7 + 3, HiDAC, and PBSCT (Table 4). Two of the 5 objective responders had no prior ARA-C exposure, whereas 3 (Patient ID#’s 106-004, 106-008, and 107-003) had previous 7 + 3 therapy. One patient who had failed 7 + 3 also had failed subsequent HiDAC (Patient ID#106-004). 

**Figure 1:**
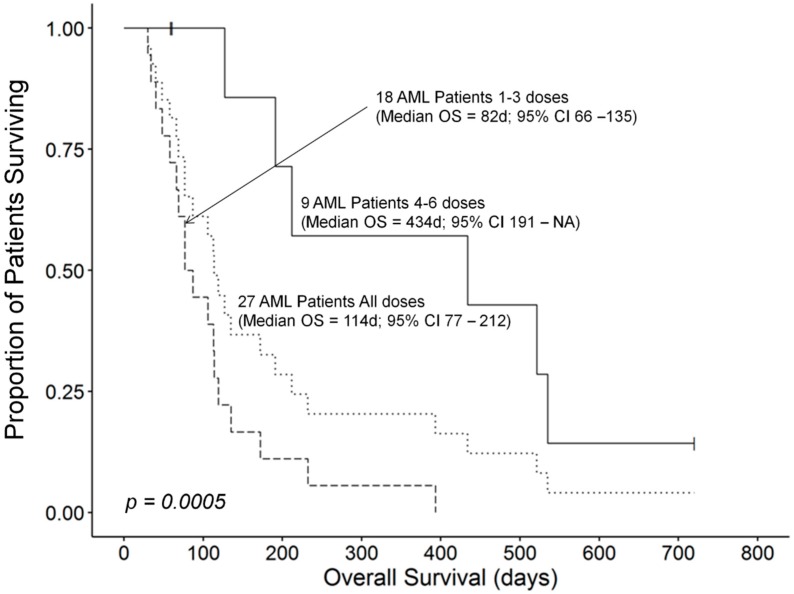
Higher number of OXi4503 doses administered is associated with improved survival outcome. The median OS time for 18 patients receiving 1–3 doses of OXi4503 was 82 (95% CI: 66–135) days and these patients exhibited a worse survival outcome compared to 9 patients receiving 4–6 doses which was recorded at 434 (95% CI: 191–NA) days (Log Rank χ^2^ = 12.3, *p*-value = 0.0004).

**Figure 2:**
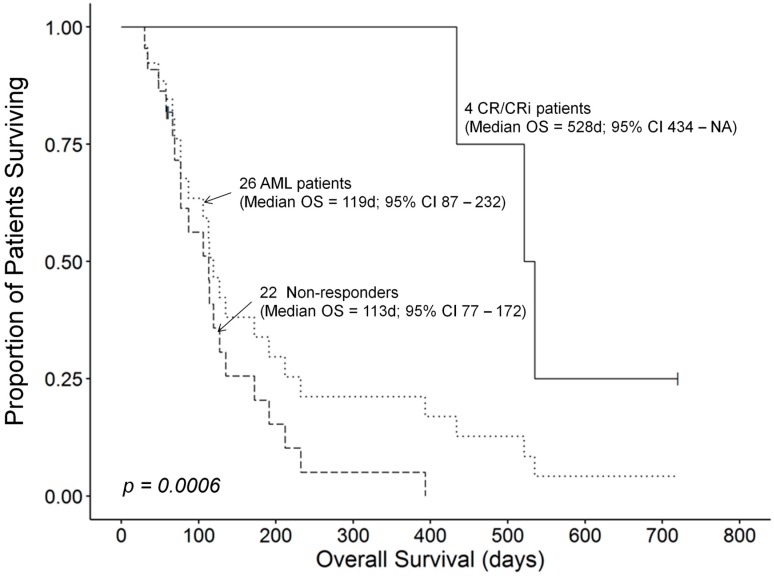
CR/CRi response to OXA is associated with improved survival outcome. The median OS time for 4 patients who achieved a CR or CRi was 528 (95% CI: 434–NA) days. By comparison, the median OS time was 113 (95% CI: 77–172) days for 22 patients who failed to achieve a CR or CRi. This difference in survival outcome was statistically significant (Log Rank χ^2^ = 11.8, *p*-value = 0.0006).

The median OS for all 26 AML patients who received therapy was 119 (95% CI: 87–232) days. Patients who had rapidly progressive diseases or developed toxicity did not receive as many OXi4503 doses as patients who responded to their treatment favorably. The median OS time for 18 patients receiving 1–3 doses of OXi4503 was 82 (95% CI: 66–135) days and these patients exhibited a worse survival outcome compared to 9 patients receiving 4–6 doses which was recorded at 434 (95% CI: 191–NA) days (Log Rank χ^2^ = 12.3, *p*-value = 0.0004) (Figure 1). However, this apparent dose effect could be biased by comorbidities of patients as confounders in these small subgroups contributing to the observed differences in survival outcomes. A randomized study will be required to validate that number of OXi4503 doses has a significant effect on the survival outcome of R/R AML patients receiving OXA as salvage therapy. One patient who achieved PR as best response died at 61 days due to invasive fungal infection. CR/CRi responses were associated with prolonged overall survival compared to the median OS time. A 68 years old patient (Patient ID: 107-003) with relapsed AML, who had previous 7 + 3 therapy became eligible for and underwent allogeneic PBSCT, remains alive and in continuous CR at 720 days. The overall survival times in the remaining 3 CR patients were 434 days (Subject 103-009), 521 days (Subject 106-006), and 535 days (Subject 106-004), respectively (Table 4). The median OS time for the 4 patients who achieved a CR/CRi was 528 (95% CI: 434–NA) days, which was significantly better than the median OS time of 113 (95% CI: 77–172) days for the remaining 22 AML patients who did not achieve a CR (Log Rank χ^2^ = 11.8, *p*-value = 0.0006) (Figure 2).

Notably, 3 of the 4 CR/CRi responses were achieved in older relapsed AML patients ≥65 years of age. One patient had treatment-related AML (tAML). Two of the 4 CR/CRi patients had 5q- with adverse cytogenetic features (co-occurring with either inv3 or 7q-), 1 had a normal karyotype, and 1 had inv16. Of note, 3 of the 4 CR/CRi patients had a low blast burden at initiation of OXA therapy, none had leukocytosis, and 2 had MDS-related cytogenetic abnormalities. None of the 5 MLL-R^+^ patients (Patient ID#’s 107-002, 103-002, 101-002, 107-004, 103-013) responded to OXA; these patients died rapidly from disease progression with a median OS time of 28 days.

## 3. Discussion

The greatest challenge in AML is relapsed or refractory (*R*/*R*) disease [2,7,8,11,12,13,14,15,16]. For R/R AML or MDS patients, there is no consensus on a single re-induction regimen. High allele frequencies of certain somatic mutations, such as in IDH1 or IDH2, may signal an opportunity for small molecule enzyme inhibitors. However, for the majority of R/R AML or MDS patients, guideline-directed standard of care suggests enrollment in a clinical trial or empiric use of one of the many cytotoxic re-induction regimens. Although progress has been made in understanding cell-intrinsic drivers of AML and new drugs have been developed for targeting select somatic mutations, the majority of relapsed AML patients die of leukemia [2,7,8,11,12,13,14,15,16,51,52,53]. In patients at second or third relapse, the median overall survival is approximately 3 months, warranting the identification of new molecular targets and development of novel therapies aimed at overcoming the drug resistance at relapse. OXA is a novel combination therapy that consists of investigational anti-AML drug OXi4503 and intermediate dose ARA-C. The primary goal of the present study was to evaluate the safety and tolerability of OXA in patients with R/R AML.

Answering the primary purpose of this clinical Phase IB study, a dose level of 9.76 mg/m^2^ was identified as the MTD and the recommended Phase 2 dose level (RP2D) of OXi4503 when it is administered in combination with fixed doses of ARA-C 1 g/m^2^. Furthermore, this study provided the first clinical insights regarding the safety profile of the OXA regimen in R/R AML patients. Patients treated with OXA experienced neutropenic fever, pneumonia, pneumonitis with associated respiratory failure, subclinical disseminated intravascular coagulation, and myelosuppression. However, most AEs were medically manageable and OXA could safely be administered to heavily pre-treated R/R AML patients, including those with advanced age. The OXA regimen also produced early evidence of clinical efficacy. In 26 evaluable AML patients, there were 4 CRs (2 CRs and 2 CRi). One patient with CRi as the best overall response (Patient ID: 106-004) had failed 5 previous regimens, including 2 ARA-C-based regimens (7 + 3, HiDAC) as well as PBSCT. The CR responses were associated with >1-year overall survival times. One patient (Patient ID: 107-003) who achieved a CR and became eligible for allogeneic PBSCT after OXA remains alive and in continuous CR for over 720 days. The safety, feasibility, and early clinical activity of OXA in R/R AML deserves further clinical validation. Future investigation may include adaptive dose-ranging Phase 2 clinical studies. Having established the MTD/RP2D of OXi4503 for its combined use with ARA-C, we are also considering testing the OXA regimen in frontline setting in patients with high risk and relapsed AML as a prelude to hematopoietic stem cell transplantation (HSCT). We postulate that a higher quality remission with a lower MRD burden can be achieved with ARA-C-based regimens when OXi4503 is included in the regimen. A randomized study will be required in order to isolate the contribution of OXi4503 to the clinical activity of the OXA regimen.

Older patients with newly diagnosed AML respond poorly to standard induction chemotherapy and have a disappointingly poor survival outcome. For newly diagnosed older AML patients, new treatment regimens have been developed in recent years, such the combination of the BCL-2 inhibitor venetoclax with hypomethylating agents (HMA) (e.g., azacytidine/AZA and decitabine/DAC) or the combination of the Hedgehog pathway inhibitor glasdegib with LDARAC both of which showed significant clinical activity with reduction in the risk of death in randomized Phase II clinical trials [54,55]. Older patients with relapsed AML have a dismal prognosis and are in urgent need for new salvage treatment strategies for their chemo-resistant leukemia [8,56]. Many of these patients are not transplant eligible due to age- and disease-related comorbidities/frailty as well as cumulative organ toxicity from previous chemotherapy. The novel OXA combination therapy was generally well tolerated in the older adults with relapsed AML with manageable toxicity and a promising benefit to risk profile. Four of the 5 objective responders were in the ≥65-years poor prognosis age category with adverse cytogenetic features. Pending further validation in a larger patient population, these early findings indicate that OXA may have clinical impact potential as a salvage regimen for older patients with R/R AML.

Combretastatins are phenolic-stilbene natural products that bind to the colchicine binding site of tubulin and exhibit anti-mitotic as well as anti-angiogenic/vascular disrupting activity and cytotoxicity [57,58]. The synthetic combretastatin derivative OXi4503 is the phosphate pro-drug of combretastatin A-1 (OXI4531) with potent nanomolar cytotoxicity/anti-proliferative activity against leukemia cells and has been shown to disrupt the BMEC support for AML clones [41,42,43,44,45,46,47,48]. Wnt signaling has been implicated in AML leukemogenesis as well as maintenance of AML stem cells, and several mutations in AML blast cells have been associated with upregulation of Wnt signaling [59,60]. Notably, OXi4503 has been shown to inhibit the Wnt/β-catenin pathway [61].

Recent studies have identified drugs that are capable of enhancing the in vivo anti-neoplastic activity of combretastatins [62]. For example, the allosteric mTOR inhibitor temsirolimus significantly enhanced the anti-cancer activity of CA4-nanoparticles in a breast cancer model [62]. This is particularly relevant because temsirolimus is an active agent against AML progenitor cells (including leukemia-initiating cells) both in vitro and in vivo when used in combination with the NF-κB inhibitor parthenolide (PTL), a naturally occurring small molecule [63] or clofarabine [64]. Notably, temsirolimus plus low dose clofarabine (20 mg/m^2^) was evaluated as salvage therapy in older patients with AML and an overall remission rate of 21% (8% CR, 13% CRi) was reported for 53 evaluable patients [65]. We hypothesize that the addition of temsirolimus to OXA may enhance its anti-leukemic potency in older patients with relapsed AML and we are considering a pilot feasibility and proof-of-concept study. It is also noteworthy, that combretastatin A-4 phosphate has been shown to improve the potency of CAR-T cells in solid tumors, owing to its VDA activity that can improve the ability of chimeric antigen receptor (CAR)-T cells to infiltrate solid tumors [66]. No studies have yet explored if OXi4503 could potentiate the anti-AML function of anti-IL3Rα/CD123 CAR-T cells [67].

## 4. Materials and Methods

### 4.1. Investigational Medicinal Product

OXi4503[3-methoxy-6-[(1*Z*)-2-(3,4,5-trimethoxyphenyl)ethenyl]benzene-1,2-diol bis(dihydrogen phosphate monopotassium salt] (molecular formula = C_18_H_20_O_12_P_2_K_2_; molecular weight = 568.49 g/mol) is a water-soluble prodrug of cis-combretastatin A1 (OXi4500). OXi4503 for injection was supplied by Mateon Therapeutics (Agoura Hills, CA, USA) as 20 mg/vial (as the free acid) as a sterile, freeze-dried (lyophilized), white to off-white, whole or fragmented cake. The drug product is packaged in a 10-mL, Type I amber glass vial, with 20 mm Flurotec^®^ stoppers and aluminum flip-off seal. The drug product was diluted in up to 150 mL 0.9% NaCl. The intravenous infusion set (i.e., infusion bag, intravenous tubing) had to be protected from light.

### 4.2. Patients and Patient Disposition

38 candidates were screened; of which, 8 candidates were screening failures, and one candidate was treated with single agent OXi4503. The remaining 29 patients were enrolled and treated according to study protocol. To be eligible for the study, patients had to be ≥18 years of age with AML (de novo or secondary, and any World Health Organization (WHO) 2008 classification excluding acute promyelocytic leukemia) that failed to achieve complete remission (CR) or morphologic complete remission with incomplete blood count recovery (CRi) (International Working Group [IWG] 2003) after at least 1 cycle of induction chemotherapy, or relapsed after any duration of any hematologic response; or MDS with marrow blasts >5% and disease failed at least 1 prior hypomethylating agent (IWG 2006). Eligibility required an Eastern Cooperative Oncology Group (ECOG) performance status 0, 1, or 2; total bilirubin ≤ 2, except in the setting of Gilbert’s disease or hemolysis; serum aspartate aminotransferase (AST) and alanine aminotransferase (ALT) levels ≤ 2.5 times upper limit of normal (ULN); serum creatinine < 2.5 times ULN; and prothrombin time (PT)/international normalized ratio (INR) and partial thromboplastin time (PTT) in normal range ± 25%. Exclusion criteria included diagnosis of acute promyelocytic leukemia (APL) with t(15;17); absolute peripheral blood myeloblast count greater than 20,000/mm^3^; uncontrolled hypertension, defined as blood pressure ≥140/90 mm Hg despite maximum medical intervention; history of congenital long QT syndrome or Torsades de pointes; pathologic bradycardia or heart block (excluding first degree heart block, prolonged baseline QTc, defined as QTcF (Fridericia correction) interval >480 msec (including subjects with a bundle branch block; history of ventricular arrhythmia (excluding premature ventricular contractions [PVCs]); major surgery within 28 days; unstable angina pectoris within 28 days, myocardial infarction and/or new ST elevation or depression or new Q wave on electrocardiogram (ECG) within 28 days; any history of hemorrhagic stroke, symptomatic congestive heart failure Class III or greater (New York Heart Association Functional Classification); use of full dose anti-coagulation; major hemorrhagic event within 28 days requiring transfusion of packed red blood cells; prior history of hypertensive crisis or hypertensive encephalopathy; clinical evidence suggestive of central nervous system (CNS) involvement with leukemia unless a lumbar puncture was performed to confirm the absence of leukemic blasts in the cerebrospinal fluid (CSF); systemic fungal, bacterial, viral, or other infection not controlled (defined as exhibiting ongoing signs/symptoms related to the infection and without improvement, despite appropriate antibiotics or other treatment); any open wound; pregnancy/lactation; and treatment with any anticancer therapy (standard or investigational) within the previous 14 days prior to the first dose of study drug. In addition, subjects had to have fully recovered (National Cancer Institute Common Terminology Criteria for Adverse Events [NCI CTCAE] Grade 1) from the clinically significant toxic effects of previous therapy. The use of hydroxyurea in subjects with rapidly proliferating disease was allowed only during Cycle 1 for up to 2 weeks after first dosing in Cycle 1 (e.g., Days 1–14). Baseline and follow-up laboratory tests were performed according to standard methods in CLIA-certified laboratories. Molecular profiling was performed using the Genoptix (Carlsbad, CA, USA) platform that utilizes DNA sequencing to interrogate 21 genes known to be recurrently mutated in AML.

### 4.3. Study Conduct

In each cohort of this multi-institutional, open-label, dose-finding Phase IB study (NCI-2016-00143; sponsor designation: OXI1222), eligible AML/MDS patients were assigned to receive OXi4503 in combination with ARA-C (OXA). OXi4503 was intravenously (IV) infused over 10 min on Days 1 and 4 of a 28-day cycle. Fixed doses of ARA-C 1 g/m^2^ were infused IV over 2 h daily on Days 1–5 of the 28-day cycle. On Days 1 and 4, OXi4503 was administered 4 h prior to the ARA-C infusions. This trial was registered at www.clinicaltrials.gov as NCT02576301.

Four centers in the US recruited patients under approval by an Institutional Review Board (IRB) in accordance with the Declaration of Helsinki. All patients provided informed consent before administration of any study treatment. The starting dose of OXi4503 in the OXA regimen was 3.75 mg/m^2^. Seven patients each received a dose of 3.75 mg/m^2^ and 9.76 mg/m^2^ OXi4503 in Cohort 1 and 5, respectively. Four patients each received 4.68 mg/m^2^, 6.25 mg/m^2^, and 12.2 mg/m^2^ OXi4503 in Cohorts 2, 3 and 6, respectively. Three patients received 7.81 mg/m^2^ OXi4503 in Cohort 4. Table S1 lists all the 29 patients who received OXA by dose cohorts.

Patients on OXA who tolerated Cycle 1 of induction treatment, and did not have progressive disease or dose-limiting adverse events were eligible to continue to receive a second induction cycle of OXA treatment. Additional cycles of OXA therapy at the same dose levels of OXi4503 and ARA-C could be given for a total of 4 cycles unless there was disease progression, unacceptable toxicity, or consent withdrawal.

The primary endpoint was the determination of the MTD of OXi4503 in combination with 1 g/m^2^/day ARA-C in participants with R/R AML or MDS. The MTD for OXi4503 was defined as the highest dose level at which <2 of 3–6 subjects, <3 of 7–9 subjects, <4 of 10–12 subjects, or <5 of 13–15 subjects experience a dose limiting toxicity (DLT) A DLT for OXA was defined as: (i) Any Grade ≥3 drug-related non-hematologic toxicity, with the following exceptions: Grade 3 diarrhea, nausea or vomiting adequately controlled with supportive measures to <Grade 2 within 72 h; Grade 3 mucositis which resolves to ≤Grade 2 within 1 week; Grade 3 infection or febrile neutropenia adequately controlled with supportive antimicrobials (defervescence within 48 h, hemodynamic stability, no growth from blood cultures, no signs of sepsis); Grade 3–4 electrolyte imbalance that corrects within 48 h to ≤Grade 2; Isolated elevations in transaminases in the absence of a concomitant rise in bilirubin which resolves to ≤Grade 2 within 7 days; Grade 3–4 fatigue ≤ 7 days in duration; (ii) Grade 4 neutropenia and/or thrombocytopenia (thought to be due to marrow hypoplasia and not leukemic burden) that does not recover to <Grade 3 within 6 weeks (bone marrow aspirate/biopsy will be required to confirm a hypocellular marrow); (iii) Grade 3 or 4 laboratory indices of coagulation abnormalities with clinical evidence of hemorrhage. For the purpose of dose escalation/dose reduction decisions, only DLTs that occurred during Cycle 1 were considered. This was due to the fact that cumulative toxicities were not observed in previous Phase IA studies of OXi4503, and the plasma half-lives of OXi4503 and its active metabolite OX4500 were determined to be only 1.7 h and 5.0 h, respectively. All participants were allowed to be premedicated with standard anti-emetic therapy per American Society of Clinical Oncology (ASCO) guidelines. Secondary endpoints included the assessment of the safety and tolerability of OXi4503 when used in combination with 1 g/m^2^/day ARA-C, and assessments of clinical anti-leukemic activity and survival times of patients treated with OXA. Outcome measurements for safety included evaluation of DLTs, incidence of adverse events (AEs), including serious adverse events (SAEs), change in clinical laboratory tests (serum chemistry and hematology), change in vital signs, change in physical examination parameters, change in ECGs, and change in concomitant medication use. Adverse event assessments, physical examinations, complete blood counts, chemistry panel, vital signs and ECOG performance status were documented every week for the duration of the clinical trial. Safety was assessed from the day of informed consent to 30 days after final dose of OXi4503. AEs were recorded for all patients that received at least one dose of OXi4503 and one post-dose safety assessment. AEs were graded according to the NCI CTCAE version 4.0. Investigators assessed the causality of AEs as either unrelated, possibly, probably, or definitely related to OXi4503 treatment.

The efficacy outcome of interest was objective response (OR) in the AML and MDS patient populations, defined as CR, CRi, or PR, based on the IWG for Diagnosis, Standardization of Response Criteria, Treatment Outcomes, and Reporting Standards for Therapeutic Trials in AML (2003), and as CR, marrow CR, or PR in MDS (IWG 2006). However, only two MDS patients were enrolled and therefore, efficacy evaluations have focused on AML. Patients who completed Cycle 1 were evaluated for response; however, patients who did not complete Cycle 1, but were considered to have PD per the investigator’s discretion, were also considered evaluable. Patients who died for any reason other than progressive disease or withdrew consent prior to first bone marrow (BM) assessment were considered non-evaluable (NE) for efficacy.

### 4.4. Ethics Statement and Study Approval

The protocol was approved by the IRB or independent ethics committee at each participating center and the study was performed in accordance with the Declaration of Helsinki, the International Conference on Harmonization (ICH)–Good Clinical Practice guidelines, and local laws. Each patient provided a written informed consent. The IRB-approved study/protocol numbers were #20152373 for Western Institutional IRB (University of Florida), #16-000292 (UCLA), #e20150655 (University of Miami Sylvester Comprehensive Cancer Center), and #00003368 (University of Kansas Cancer Center).

### 4.5. Statistical Analyses

Standard statistical methods were applied for the analysis of data. The distribution of time-to-event survival end points on the OS and PFS curves were estimated by the Kaplan–Meier method. Differences between patient subgroups were evaluated by log-rank statistics. The analyses were performed using JMP software (version 10.02, SAS Institute, Inc., Cary, NC, USA), and R software version 3.5.2 (R Foundation for Statistical Computing, Vienna, Austria) loaded with the survival_2.44-1.1 statistical package for survival analysis [68] and its extensions survMisc_0.5.5 (https://CRAN.R-project.org/package=survMisc) and survminer_0.4.4 (https://CRAN.R-project.org/package=survminer).

## 5. Conclusions

Our Phase 1B clinical study of combretastatin A1 diphosphate (OXi4503) and ARA-C in combination (OXA) shows that this novel combination therapy is generally well tolerated by R/R AML patients with an OXi4503 MTD of 9.76 mg/m^2^. In 26 evaluable AML patients, there were 2 CR, 2 CRi and one PR. The CR/CRi responses were associated with >1-year overall survival times. The novel OXA combination therapy was generally well tolerated in the older adults with relapsed AML with manageable toxicity and a promising benefit to risk profile. Four of the 5 objective responders were in the unfavorable age category of ≥65-years-old and unfavorable cytogenetics. The safety, feasibility, and early clinical activity of OXA in R/R AML deserves further clinical validation. A randomized study will be required in order to isolate and fully appreciate the contribution of OXi4503 to the clinical activity of the OXA regimen.

## Figures and Tables

**Table 1:** Patient Characteristics and Demographic Features for Safety.

Diagnosis	Number of Patients (or Years–for Age Only)
AML	27 (93.1%)
MDS	2 (6.9%)
**Age** (years)	
Mean ± SE	57.8 ± 2.6
Median	61
Range	26–78
**Sex**	
Female	12 (41.4%)
Male	17 (58.6%)
**Ethnic Origin**	
Caucasian	21 (72.4%)
Black or African American	3 (10.3%)
Hispanic	2 (6.9%)
Other/Not reported	3 (10.3%)
**Prior # of Chemotherapy Regimens**	
1	4
2	12
3	4
≥4	8
Not reported	1
**No of OXi4503 Doses**	
1	2
2	18
>4	9

Population treated with combretastatin A1 diphosphate (OXi4503) and cytarabine (ARA-C) in combination (OXA) (*N* = 29). The median number of OXi4503 doses administered per patient was 2 (Range = 1–6).

**Table 2:** Incidence of OXi4503-related Grade 3–4 AEs (adverse events) occurring in study OX1222 patients treated with OXA—by MedDRA PT.

MedDRA SOC MedDRA PT	Cohorts (Number of Patients/Cohort) *n* (%)						Total
	3.75 mg/m^2^ (7)	4.68 mg/m^2^ (4)	6.25 mg/m^2^ (4)	7.81 mg/m^2^ (3)	9.76 mg/m^2^ (7)	12.2 mg/m^2^ (4)	(*N* = 29) *n* (%)
**Blood and Lymphatic System Disorders**							
**Anemia**	**1 (14.3)**	**1 (25)**	**0**	**1 (33.3)**	**1 (14.3)**	**0**	**4 (13.8)**
Grade 3	0	1 (25)	0	1	1(14.3)	0	3 (10.3)
Grade 4	1 (14.3)	0	0	0	0	0	1 (3.4)
**Blood Bilirubin Increased**	**0**	**0**	**0**	**0**	**0**	**1 (25)**	**1 (3.4)**
Grade 3	0	0	0	0	0	1(25)	1 (3.4)
**Febrile Neutropenia**	**0**	**1 (25)**	**2 (50)**	**3 (100)**	**1 (14.3)**	**1 (25)**	**8 (27.6)**
Grade 3	0	1(25)	2 (50)	3 (100)	1 (14.3)	1 (25)	8 (27.6)
**Neutropenia**	**0**	**1 (25)**	**0**	**0**	**0**	**0**	**1 (3.4)**
Grade 4	0	1(25)	0	0	0	0	1 (3.4)
**Thrombocytopenia**	**0**	**1 (25)**	**0**	**0**	**0**	**0**	**1 (3.4)**
Grade 4	0	1 (25)	0	0	0	0	1 (3.4)
**Infections and Infestations**							
**Bacteremia**	**0**	**0**	**0**	**1 (33.3)**	**0**	**0**	**1 (3.4)**
Grade 3	0	0	0	1(33.3)	0	0	1 (3.4)
**Pneumonia**	**0**	**0**	**0**	**0**	**0**	**1 (25)**	**1 (3.4)**
Grade 3	0	0	0	0	0	1 (25)	1 (3.4)
**Investigations**							
**Aspartate Aminotransferase Increased**	**1 (14.3)**	0	0	0	0	**1 (25)**	**2 (6.9)**
Grade 3	1 (14.3)	0	0	0	0	1 (25)	2 (6.9)
**Blood Bilirubin Increased**	0	0	0	0	0	**1 (25)**	**1 (3.4)**
Grade 3	0	0	0	0	0	1 (25)	1 (3.4)
**Blood Fibrinogen Decreased**	**1 (14.3)**	0	0	0	0	0	**1 (3.4)**
Grade 3	1 (14.3)	0	0	0	0	0	1 (3.4)
**Neutrophil Count Decreased**	**2 (28.6)**	0	0	**1 (33.3)**	**1 (14.3)**	0	**4 (13.8)**
Grade 3	1 (14.3)	0	0	0	1 (14.3)	0	2 (6.9)
Grade 4	1 (14.3)	0	0	1 (33.3)	0	0	2 (6.9)
**Platelet Count Decreased**	**1 (14.3)**	**1 (25)**	0	**1 (33.3)**	**1 (14.3)**	0	**5 (17.2)**
Grade 3	0	0	0	0	1(14.3)	0	1 (3.4)
Grade 4	2 (28.6)	1 (25)	0	1 (33.3)	0	0	4 (13.8)
**Prothrombin Time Prolonged**	**1 (14.3)**	0	0	0	0	0	**1 (3.4)**
Grade 3	1 (14.3)	0	0	0	0	0	1 (3.4)
**White blood Cell Count Decreased**	0	0	0	**1 (33.3)**	**1 (14.3)**	0	**2 (6.9)**
Grade 4	0	0	0	1 (33.3)	1 (14.3)	0	2 (6.9)
**Metabolism and Nutrition Disorders**							
**Hypokalemia**	**1 (14.3)**	0	0	0	0	0	**1 (3.4)**
Grade 3	1 (14.3)	0	0	0	0	0	1 (3.4)
**Respiratory, Thoracic and Mediastinal Disorders**							
**Respiratory Failure**	0	0	0	0	0	**1 (25)**	**1 (3.4)**
Grade 4	0	0	0	0	0	1 (25)	1 (3.4)
**Vascular Disorders**							
**Hypertension**	**1 (14.3)**	0	0	**1 (33.3)**	**1 (14.3)**	**2 (50)**	**5 (17.2)**
Grade 3	1 (14.3)	0	0	1 (33.3)	1 (14.3)	2 (50)	5 (17.2)
**Hypotension**	0	0	0	0	0	**1 (25)**	**1 (3.4)**
Grade 3	0	0	0	0	0	1 (25)	1 (3.4)

Multiple events for the same term and patient have been reported as 1 event only, unless same event was reported for 2 different Grades i.e., worsened Depicted are patient numbers in each cohort and their percentage experiencing Grade 3–4 AE as well as the total number of the Grade 3–4 AE and their percentage in the entire patient population across all dose cohorts.

**Table 3:** OXi4503-related serious adverse events (SAE).

A All Study Drug OXi4503-Related SAEs Reported for Patients Treated with OXA in Study OX1222.								
Patient No.	Cohort#	SAE Reported Term (CTCAE Grade)		Relatedness with OXi4503		SAE Outcome		Action Taken
106–006	2	Neutropenic Fever (3)		Possibly related		Recovered, without sequelae		None
103–010	4	Neutropenic fever (3)		Related		Recovered, without sequelae		None
103–012	6	Acute hypoxic respiratory failure (4)		Possibly related		Recovered		None
103–012	6	Pneumonia (3)		Possibly related		Not recovered		None
106–011	6	Hypotension (3)		Possibly related		Recovered, without sequelae		None
**B Incidence of OXi4503-related SAEs Occurring in patients treated with OXA in study OX1222 by MedDRA PT (any CTCAE Grade).**								
**MedDRA SOC** **MedDRA PT**		**Cohorts**						**Total**
		**3.75 mg/m^2^**	**4.68 mg/m^2^**	**6.25 mg/m^2^**	**7.81 mg/m^2^**	**9.76 mg/m^2^**	**12.2 mg/m^2^**	***N* = 29** ***n*(%)**
**Blood and Lymphatic System Disorders**								
Febrile Neutropenia		0	1	0	1	0	0	2 (6.9)
**Respiratory, Thoracic and Mediastinal Disorders**								
Acute hypoxic Respiratory Failure		0	0	0	0	0	1	1 (3.4)
Pneumonia		0	0	0	0	0	1	1 (3.4)
**Vascular Disorders**								
Hypotension		0	0	0	0	0	1	1 (3.4)

**Table 4:** Relapsed AML Patients Who Had an Objective Response to OXA in OX1222 Study (*N* = 5).

							Bone Marrow Involvement		Treatment Outcome						
Patient ID	Cohort	OXi4503 Doses	Diagnosis	Age/Sex/Race	Previous Therapies (Number: List)	Cellularity	Percent Myeloblasts by Morphology/FCM	Karyotype/Mutations	Best Overall Response	Time to Progression (days)	C1D1 to EOS	Other Therapy post EOS	Time to Death or Last FU	Survival Status at lastFU	Cause of Death
106-004 ^1^	1	4	AML	59/M/C	5: 7 + 3; HiDAC;5-AC; IP; PBSCT	NR	25/20	Inv (3) (q21q26.2), del(5)(q)/PTPN11 (9%VAF)	CRi	54+	54	DLI	535	D	PD
106-006 ^2^	2	4	AML	65/M/C	1: 5AZA	20–30	9/8	46, XY, +8 (FISH)//NR	CRi	64+	64	Mylotarg 5-AC	521	D	PD
103-009 ^3^	3	4	tAML	66/M/C	1: 5-AC+DAUNO	20–70	15	del(5)(q), del(7)(q), +8 (FISH)/TP53	CR	78+	78	5AZA	434	D	PD
106-008 ^4^	4	4	AML	77/F/C	2: 7 + 3; 5AZA	80–90	89/65	46, XX	PR	NA	61	NA	61	D	IFI
107-003 ^5^	5	6	AML	68/M/C	1: 7 + 3	60	15/9	Inv (16) (p13q22) (FISH)/CBFB	CR	228+	NA	Allo PBSCT	720	A	NA

C1D1: Cycle 1 Day 1; FU: Follow-up; AML: Acute myelogenous leukemia; MDS: Myelodysplastic syndromes; PD: Progressive disease; CRi: Complete remission with incomplete hematologic recovery; PR: Partial remission; SD/RES: Stable disease/Refractory; NE: Not evaluable; CR: Complete remission; A: Alive; D: Dead; EOS: End of study; NA: Not available; SAE: Serious adverse event; HU: Hydroxyurea; DLI: Donor leukocyte infusion; 5-AC: 5-Azacytidine; PBSCT: Peripheral blood stem cell transplantation; SCT: Stem cell transplant; 5AZA: Deoxyazacytidine. IFI: Invasive fungal infection. ^1^ The BM blast percentage went from 25% to 3% microscopically and 20.1% to 0.2% by FCM after one cycle of OXA with clearance of PTPN11. ^2^ The BM blast percentage went from 9% to 2% microscopically and from 8.1% to 0.3% by FCM after 2 cycles of OXA. ^3^ The BM blast percentage went from 15% to 2% microscopically and genomic missense mutations in the TP53 gene (c.428T>G; p.V143G; 9% allele frequency) detected by molecular profiling using the Genoptix platform cleared after 2 cycles of OXA. ^4^ The BM blast percentage went from 89% to 7% microscopically and from 65% to 2% by FCM after one cycle of OXA. ^5^ The BM percentage went from 15% to 0% microscopically and from 9% to 1–2% by FCM with disappearance (0 of 300 nuclei) of the FISH-detected split signal (10%/30 of 300 nuclei) pre-therapy) due to inv(16)/CBFB rearrangement after one cycle of OXA.

## Supplementary Materials

The following are available online at https://www.mdpi.com/2072-6694/12/1/74/s1. Table S1: OX1222 Phase I Study Patients Treated with OXi4503 in combination with Cytarabine (OXA) according to dose cohorts of OXi4503, Table S2: Patient Characteristics and Demographic Features for Safety Population Treated with OXA in OX1222 Study (*N* = 29), Table S3: Incidence of adverse events of all grades by MedDRA PT (any CTCAE Grade) occurring in patients treated with OXA in Study OX1222 regardless of any relationship with the study drug OXi4503, Table S4: Incidence of adverse events CTCAE Grade 3-5 by MedDRA PT occurring in patients treated with OXA in Study OX1222 regardless of any relationship with the study drug OXi4503, Table S5: All Severe Adverse Events (SAEs) Occurring After Treatment with OXA in the OX1222 study, Table S6: Incidence of SAEs occurring after treatment with OXA by MedDRA PT (any CTCAE Grade) regardless of any relationship with the study drug OXi4503, Table S7: Incidence of laboratory abnormalities* CTCAE Grade 3–4 by MedDRA PT regardless of any relationship with the study drug OXi4503, Table S8: Incidence of OXi4503-related AEs by MedDRA PT (any CTCAE Grade), Table S9: OXi4503-related Grade 3/4 AEs and Survival Outcome in Study OX1222 patients treated with OXA, Table S10: All study drug-related Grade 3–4 adverse events* reported in Study OX1222.
