# Supplementary material for: A Phase 1B Clinical Study of Combretastatin A1 Diphosphate (OXi4503) and Cytarabine (ARA-C) in Combination (OXA) for Patients with Relapsed or Refractory Acute Myeloid Leukemia
Source: Cancers (Basel). 2019 Dec 26;12(1):74. doi: 10.3390/cancers12010074 (PMC7016810; doi:10.3390/cancers12010074)
Supplement: Supplementary file 1 [file cancers-12-00074-s001.pdf]

Supplementary Materials

A Phase 1B Clinical Study of Combretastatin A1 Diphosphate (OXi4503) and Cytarabine (ARA-C) in Combination (OXA) for Patients with Relapsed or Refractory Acute Myeloid Leukemia

Fatih M. Uckun, Christopher R. Cogle, Tara L. Lin, Sanjive Qazi, Vuong N. Trieu and Gary Schiller, Justin Watts

Table S1: OX1222 Phase I Study Patients Treated with OXi4503 in combination with Cytarabine (OXA) according to dose cohorts of OXi4503.

	Cohorts					
	**1**	**2**	**3**	**4**	**5**	**6**
	**3.75 mg/m^2**	**4.68 mg/m^2**	**6.25 mg/m^2**	**7.81 mg/m^2**	**9.76 mg/m^2**	**12.2 mg/m^2**
	101-002	103-005	101-005	101-006	101-007	103-012
	103-002	103-008	103-009	103-010	103-011	103-013
	103-003	106-005	107-001	106-008	106-009	106-010
	103-004	106-006	107-002		107-003	106-011
	106-001				107-004	
	106-003				107-007	
	106-004				107-008	
**Total**	**7**	**4**	**4**	**3**	**7**	**4**

Table S2. Patient Characteristics and Demographic Features for Safety Population Treated with OXA in OX1222 Study (*N* = 29).

						Bone Marrow Involvement			Treatment Outcome						
Patient ID	Cohort	OXi4503 Doses	Diagnosis	Age/Sex/Race	Previous Therapies (Number: List)	Cellularity	Percent Myeloblasts by Morphology/FCM	Karyotype/Mutations	Best Overall Response	Time to Progression (days)	C1D1 to EOS	Other Therapy post EOS	Time to Death or Last FU	Survival Status at last FU	Cause of death
101-002	1	2	AML	61/M/C	4: IDAC;MCC; CECA;5AZA	95	30/25	t(11;15)/MLL-R;FLT3	PD	28	28	HU	30	D	PD
106-004	1	4	AML	59/M/C	5: 7+3;HiDAC;5-AC;IP;PBSCT	NR	25/20	inv(3)(q21q26.2), del(5)(q)/PTPN11	CRi	54+	54	DLI	535	D	PD
103-004	1	2	AML	68/M/B	2: IDAC;ME	90	60/-	46,XY/NPM1,JAK2, TP53	SD/RES	106	29	NR	106	D	PD
103-002	1	2	AML	31/F/B	5: 7+3;HiDAC;ME;5AZA;CAFdA-C	NR	27/-	t(4;15),t(9;11), MLL-R/NRAS	PD	16	30	NR	77	D	PD
103-003	1	2	AML	26/F/M*	5: IDAC;7+3+2-CDA;ME;5-AC+NEXAVAR	70	40/31	t(7;12)/FLT3-ITD,WT1	PD	29	33	NR	87	D	PD
106-001	1	1	AML	64/F/C	6: 7+3;HiDAC;ALLO PSCT;URD PBSCT, 5AZA;IP	20	38/-	45-48,XX,t(1;1)/NR	PD	22	30	NR	172	D	PD
106-003	1	2	AML	61/F/C	3:7+3;MEC;5AZA	20	12	t(12;15),dic(8;11),-5,-13,-11/NR	NE	NE	4	NR/LFU	40	D	NR/ LFU
106-005	2	2	MDS/ RAEB-2	31/M/C	2: Allo PSCT;5-AC	30	9/8	t(2;5),t(5;14)/WT1	PD	24	61	7+3,HU	285	D	PD
106-006	2	4	AML	65/M/C	1: 5AZA	20-30	9/8	46,XY, +8(FISH)//NR	CRi	64+	64	Mylotarg 5-AC	521	D	PD
103-005	2	4	AML	60/M/C	4:HDAC;HiDAC;CECA;5-AC	NR	5-10	NR/IDH2	PD	57	57	CAFFdA Allo-PBSCT	212	D	PD
103-008	2	2	AML	59/M/C	5: 7+3;HiDAC;ME;5-AC;REV	90	58	del(5)(q),-21/ASXL1	SD/RES	48	33	None	48	D	PD
101-005	3	2	AML	66/M/B	2: IDAC;CECA	<5	1	del(6)(p)	PD	29	32	Jakafi	119	D	PD
103-009	3	4	tAML	66/M/C	1: 5-AC+DAUNO	20-70	15*	del(5)(q),del(7)(q)/TP53	CR	78+	78	5AZA	434	D	PD
107-001	3	6	AML	60/M/C	2: IDAC; UBC-SCT	80	90/76	Hx: del(5)(q)/ Hx: RUNX1,WT1	PD	89	96	IP	191	D	NR
107-002	3	4	AML	38/F/A	2: 7+3; ALLO-PBSCT	20-30	65/59	-7, MLL-R (FISH)/PTPN11	SD/RES	NA	59	NA	59	D	IFI
106-008	4	4	AML	77/F/C	2: 7+3; 5AZA	80-90	89/65	46, XX	PR	NA	61	NA	61	D	IFI
103-010	4	2	AML	67/M/C	3: IDAC;HiDAC;ME	90	90/-	49,XY,+4,+8,+14,Del(4)(q)/ NPM1,KIT,DNMT3A	SD/RES	135	29	5-AC, NEXAVAR	135	D	PD
101-006	4	2	AML	61/M/C	2: 5-AC;IDAC	30	10-12/16	del(5)(q)/PTPN11	SD/RES	113	29	Velcade, HU	113	D	PD
106-009	5	2	AML	74/F/C	3: IDAC;HiDAC	40	18/6	NR/NR	PD	28	35	5AZA	232	D	NR/ HOS

						Bone Marrow Involvement			Treatment Outcome						
Patient ID	Cohort	OXi4503 Doses	Diagnosis	Age/Sex/Race	Previous Therapies (Number: List)	Cellularity	Percent Myeloblasts by Morphology/FCM	Karyotype/Mutations	Best Overall Response	Time to Progression (days)	C1D1 to EOS	Other Therapy post EOS	Time to Death or Last FU	Survival Status at last FU	Cause of death
107-003	5	6	AML	68/M/C	1: 7+3	60	15/9	t(16;16)/CBFB-R,KRAS	CR	228+	NA	Allo PBSCT	720	A	NA
103-011	5	2	MDS/RCMD	78/M/C	1: 5-AC	10-30	15/20	45,XY,-7	SD/RES	298	43	LDAC	298	D	PD
101-007	5	2	AML	33/M/C	2: IDAC;CECA	20-40	50/41	t(5;11)(q35;p15)/FLT3-ITD*	PD	33	35	NR	393	D	PD
107-004	5	2	AML	56/F/C	2: 5AZA;Lipo-DAUNO-C	NR	64	del(11)(q), -7	PD	25	114	NA	114	D	PD
107-007	5	1	AML	65/F/C	2: 5AZA; Busulfan	50	20	Del(5)(q), del(21)(q) -17,del(22)(q),del(7)(q)/TP53	PD	27	69	NA	69	D	PD
107-008	5	2	AML	61/F/C	NR	NR	NR	NR/NR	PD	66	66	NA	66	D	PD
103-012	6	2	AML	54/F/H	3: 2-CDA+DAUNO;aCD33-Calicheamicin/Mylotarg+MITO; Mylotarg	50	60	NR/NR	PD	27	34	-	34	D	PD
103-013	6	2	AML	60/M/H	2: Lipo-DAUNO-C; 5AZA	60	NA	t(6;11)/BRAF,KRAS,CEBPA	PD	29	29	-	77	D	PD
106-010	6	5	AML	66/M/C	2: : Lipo-DAUNO-C 5-AC	NR	57	NR/NR	SD/RES	127	64	NR/LFU	127	D	NR
106-011	6	2	AML	41/F/-	6:Busulfan;ARA-C; M-CTX; M-C; 5AZA;HU	90	52	NR/NR	PD	18	18	NR/LFU	58	D	PD

C1D1: Cycle 1 Day 1; FU: Follow-up; AML: acute myelogenous leukemia; MDS: Myelodysplastic Syndromes; PD: Progressive disease; CR i: Complete response with incomplete hematologic recovery; PR: Partial response; SD/RES: Stable disease/Refractory; NE: Not evaluable; CR: Complete response; A: Alive; D: Dead; EOS: End of Study; NA: Not available; SAE: serious adverse event; HU: hydroxyurea; DLI: donor leukocyte infusion; 5-AC: 5-Azacytidine; PBSCT: Peripheral blood stem cell transplantation; SCT: stem cell transplant; 5AZA: deoxyazacytidine. IFI: Invasive fungal infection.

Table S3. Incidence of adverse events of all grades by MedDRA PT (any CTCAE Grade) occurring in patients treated with OXA in Study OX1222 regardless of any relationship with the study drug OXi4503.

	Cohorts						Total
**MedDRA SOC**<br>**MedDRA PT**	**3.75 mg/m²**	**4.68 mg/m²**	**6.25 mg/m²**	**7.81 mg/m²**	**9.76 mg/m²**	**12.2 mg/m²**	**N = 29**<br>**n (%)**
**Blood and lymphatic system disorders**							
Anemia	4	2	1	1	2	1	11 (37.9)
Disseminated intravascular coagulation	0	0	0	0	0	1	1 (3.4)
Febrile neutropenia (FN)	3	3	4	3	3	3	19 (65.5)
Leukocytosis	1	0	0	0	0	0	1 (3.4)
Lymph node pain	1	0	0	0	0	0	1 (3.4)
Neutropenia (does not include FN report)	0	1	0	0	1	0	2 (6.9)
Pancytopenia	1	0	1	0	0	0	2 (6.9)
Thrombocytopenia	0	1	0	0	0	0	1 (3.4)
**Cardiac disorders**							
Sinus bradycardia	1	0	0	0	0	0	1 (3.4)
Sinus tachycardia	0	0	0	1	0	0	1 (3.4)
Tachycardia	1	1	1	1	2	0	6 (20.7)
**Eye disorders**							
Blepharitis	0	0	1	0	0	0	1 (3.4)
Conjunctival hemorrhage	1	0	0	0	0	0	1 (3.4)
Dry eye	1	0	0	0	0	0	1 (3.4)
Keratitis	0	0	1	0	0	0	1 (3.4)
Eye swelling	0	0	1	0	0	0	1 (3.4)
Visual impairment	0	0	1	0	0	0	1 (3.4)
**Gastrointestinal disorders**							
Abdominal distension	0	0	1	0	0	0	1 (3.4)
Abdominal pain	1	0	1	1	0	0	3 (10.3)
Abdominal tenderness	1	0	0	0	0	0	1 (3.4)
Anal pruritus	0	0	0	1	0	0	1 (3.4)
Bowel movement irregularity	0	1	0	0	0	0	1 (3.4)
Constipation	1	2	1	0	3	0	7 (24.1)
Diarrhea	3	1	3	1	1	1	10 (34.5)
Dyspepsia	0	1	0	1	0	0	2 (6.9)
Flatulence	0	1	0	1	0	0	2 (6.9)
Gingival disorder	1	0	0	0	0	0	1 (3.4)
Hematochezia	1	0	0	0	0	0	1 (3.4)
Hemorrhoidal hemorrhage	0	0	1	0	0	0	1 (3.4)
Hemorrhoids	0	1	1	1	0	1	4 (13.8)
Large intestinal hemorrhage	0	0	0	0	1	0	1 (3.4)
Lip ulceration	0	0	1	0	0	0	1 (3.4)
Nausea	4	2	1	2	3	1	13 (44.8)
Oral pain	0	0	2	0	0	0	2 (6.9)
Pancreatitis acute	1	0	0	0	0	0	1 (3.4)

MedDRA SOC<br>MedDRA PT	Cohorts<br>3.75 mg/m²	4.68 mg/m²	6.25 mg/m²	7.81 mg/m²	9.76 mg/m²	12.2 mg/m²	Total<br>N = 29<br>n (%)
Proctalgia	0	0	1	0	0	0	1 (3.4)
Proctitis	0	1	0	0	0	0	1 (3.4)
Rectal hemorrhage	0	0	1	0	0	0	1 (3.4)
Stomatitis	1	1	0	0	1	1	4 (13.8)
Tongue ulceration	0	1	0	0	0	0	1 (3.4)
Toothache	1	0	0	0	0	0	1 (3.4)
Vomiting	3	0	1	1	2	0	7 (24.1)
**General disorders and administration site conditions**							
Asthenia	2	0	0	0	0	1	3 (10.3)
Axillary pain	1	0	0	0	0	0	1 (3.4)
Catheter site hemorrhage	1	0	0	0	0	0	1 (3.4)
Chills	0	0	3	1	2	1	7 (24.1)
Fatigue	4	1	0	1	0	0	6 (20.7)
Influenza like illness	0	1	0	0	0	0	1 (3.4)
Infusion site erythema	0	0	0	0	1	0	1 (3.4)
Infusion site pruritus	0	0	0	0	1	0	1 (3.4)
Infusion site thrombosis	0	0	0	0	1	0	1 (3.4)
Localized edema	0	0	1	0	0	0	1 (3.4)
Malaise	2	0	0	0	0	0	2 (6.9)
Edema	1	0	0	0	0	0	1 (3.4)
Edema peripheral	0	0	0	2	1	1	4 (13.8)
Pain	0	0	0	1	0	1	2 (6.9)
Peripheral swelling	1	0	0	0	0	0	1 (3.4)
Pyrexia	1	1	2	0	0	0	4 (13.8)
**Hepatobiliary disorders**							
Cholecystitis	1	0	0	0	0	0	1 (3.4)
**Infections and infestations**							
Atypical pneumonia	0	0	0	1	0	0	1 (3.4)
Bacteremia	0	0	1	1	0	0	2 (6.9)
Bacterial infection	0	2	0	0	0	0	2 (6.9)
Bronchopulmonary aspergillosis	0	0	1	0	0	0	1 (3.4)
Candida infection	0	0	1	0	0	0	1 (3.4)
Cellulitis	1	1	0	0	0	0	2 (6.9)
Clostridium difficile infection	0	0	0	0	1	0	1 (3.4)
Enterococcal infection	1	0	0	0	0	0	1 (3.4)
Escherichia bacteremia	0	1	0	0	0	0	1 (3.4)
Liver abscess	1	0	0	0	0	0	1 (3.4)
Oral herpes	0	0	0	1	0	0	1 (3.4)
Periorbital cellulitis	1	0	0	0	0	0	1 (3.4)
Perirectal abscess	0	0	0	1	0	0	1 (3.4)
Pneumonia	0	1	0	0	0	2	3 (10.3)
Pneumonia klebsiella bacteremia	0	0	0	0	1	0	1 (3.4)

MedDRA SOC<br>MedDRA PT	Cohorts 3.75 mg/m²	4.68 mg/m²	6.25 mg/m²	7.81 mg/m²	9.76 mg/m²	12.2 mg/m²	Total N = 29 n (%)
Sepsis	0	0	0	0	1	0	1 (3.4)
Sinusitis fungal	0	0	0	1	0	0	1 (3.4)
Staphylococcal infection	1	0	1	0	0	0	2 (6.9)
Streptococcal bacteremia	0	0	0	0	1	0	1 (3.4)
**Injury, poisoning and procedural complications**							
Contusion	1	0	0	0	0	0	1 (3.4)
Fall	0	0	0	1	0	0	1 (3.4)
Wound	0	1	0	0	0	0	1 (3.4)
Infusion related reaction	0	0	0	0	0	2	2 (6.9)
**Investigations**							
Activated partial thromboplastin time prolonged	1	0	0	0	0	0	1 (3.4)
Alanine aminotransferase increased	1	0	0	0	1	1	3 (10.3)
Aspartate aminotransferase increased	1	0	0	1	0	2	4 (13.8)
Blood alkaline phosphatase increased	0	0	0	0	0	1	1 (3.4)
Blood bilirubin increased	0	0	0	1	0	1	2 (6.9)
Blood creatinine increased	0	1	0	1	0	1	3 (10.3)
Blood fibrinogen decreased	2	0	0	1	1	1	5 (17.2)
Blood potassium decreased	0	0	1	0	0	0	1 (3.4)
Breath sounds abnormal	0	1	0	0	0	0	1 (3.4)
Ejection fraction decreased	0	0	0	1	0	0	1 (3.4)
Electrocardiogram QT prolonged	0	0	1	1	0	0	2 (6.9)
Fibrin D-dimer increased	0	0	0	0	1	0	1 (3.4)
International normalized ratio increased	0	1	0	1	0	0	2 (6.9)
Lipase increased	1	0	0	0	0	0	1 (3.4)
Neutrophil count decreased	2	0	0	1	1	0	4 (13.8)
Platelet count decreased	2	2	0	1	1	1	7 (24.1)
Prothrombin time prolonged	1	0	0	0	0	0	1 (3.4)
Transaminases increased	0	0	1	0	0	0	1 (3.4)
White blood cell count decreased	0	0	0	1	1	0	2 (6.9)
White blood cell count increased	1	0	0	0	0	0	1 (3.4)
**Metabolism and nutrition disorders**							
Decreased appetite	0	0	0	2	0	0	2 (6.9)
Dehydration	1	0	0	0	0	0	1 (3.4)
Fluid overload	0	0	0	0	1	0	1 (3.4)
Hyperammonemia	0	0	0	0	0	1	1 (3.4)
Hyperchloremia	1	0	0	0	0	0	1 (3.4)
Hyperglycemia	0	0	2	0	0	0	2 (6.9)
Hyperkalemia	0	1	0	0	0	0	1 (3.4)
Hypermagnesemia	1	0	0	0	0	0	1 (3.4)
Hyperuricemia	1	0	0	1	0	0	2 (6.9)

MedDRA SOC<br>MedDRA PT	Cohorts 3.75 mg/m²	4.68 mg/m²	6.25 mg/m²	7.81 mg/m²	9.76 mg/m²	12.2 mg/m²	Total N = 29 n (%)
Hypocalcemia	0	1	0	0	0	0	1 (3.4)
Hypokalemia	2	1	1	1	0	0	5 (17.2)
Hypomagnesemia	0	0	0	0	0	1	1 (3.4)
Hyponatremia	0	1	0	1	0	0	2 (6.9)
Hypophosphatemia	1	0	1	1	0	0	3 (10.3)
Metabolic acidosis	0	0	1	0	0	0	1 (3.4)
**Musculoskeletal and connective tissue disorders**							
Arthralgia	0	1	0	0	0	0	1 (3.4)
Back pain	2	1	1	1	0	0	5 (17.2)
Bone pain	0	0	2	1	1	1	5 (17.2)
Muscle spasms	0	1	0	0	0	0	1 (3.4)
Musculoskeletal stiffness	0	0	0	0	1	0	1 (3.4)
Neck pain	0	0	0	0	1	0	1 (3.4)
Pain in jaw	0	0	0	0	2	0	2 (6.9)
**Nervous system disorders**							
Cerebrovascular accident	0	0	0	1	0	0	1 (3.4)
Cognitive disorder	0	0	0	1	0	0	1 (3.4)
Dizziness	1	0	0	2	0	0	3 (10.3)
Dysarthria	0	0	0	0	0	1	1 (3.4)
Embolic stroke	0	0	0	1	0	0	1 (3.4)
Facial paresis	0	0	0	1	0	0	1 (3.4)
Headache	1	0	1	1	2	3	8 (27.6)
Hypoesthesia	0	1	0	0	0	0	1 (3.4)
Neuropathy peripheral	0	0	1	0	0	0	1 (3.4)
Tremor	0	0	0	0	0	1	1 (3.4)
Sinus headache	0	0	0	0	0	1	1 (3.4)
**Psychiatric disorders**							
Adjustment disorder with depressed mood	1	0	0	0	0	0	1 (3.4)
Anxiety	1	2	0	0	0	0	3 (10.3)
Confusional state	0	0	0	0	0	1	1 (3.4)
Delirium	0	0	0	0	0	1	1 (3.4)
Depression	0	0	0	0	1	0	1 (3.4)
Insomnia	3	1	0	0	0	0	4 (13.8)
**Renal and urinary disorders**							
Acute kidney injury	0	2	0	0	0	0	2 (6.9)
Urinary hesitation	0	0	0	1	0	0	1 (3.4)
Urinary incontinence	0	0	0	0	0	1	1 (3.4)
Urinary retention	0	0	0	1	0	0	1 (3.4)
**Respiratory, thoracic and mediastinal disorders**							
Atelectasis	0	0	0	1	0	0	1 (3.4)
Cough	1	1	0	1	0	0	3 (10.3)
Dyspnea	0	1	1	0	0	0	2 (6.9)
Dyspnea exertional	1	0	0	1	0	0	2 (6.9)

MedDRA SOC<br>MedDRA PT	Cohorts<br>3.75 mg/m^2	4.68 mg/m^2	6.25 mg/m^2	7.81 mg/m^2	9.76 mg/m^2	12.2 mg/m^2	Total<br>N = 29<br>n (%)
Epistaxis	1	0	0	1	2	0	4 (13.8)
Hemoptysis	0	0	0	1	0	0	1 (3.4)
Hypoxia	0	0	1	0	1	1	3 (10.3)
Nasal congestion	0	0	1	0	0	0	1 (3.4)
Oropharyngeal pain	0	1	1	0	1	0	3 (10.3)
Pleural effusion	0	1	2	0	0	0	3 (10.3)
Pulmonary mass	0	0	1	0	0	0	1 (3.4)
Pulmonary edema	0	0	0	1	0	1	2 (6.9)
Pulmonary vascular resistance abnormality	0	0	1	0	0	0	1 (3.4)
Respiratory failure	0	0	0	0	0	1	1 (3.4)
Tachypnea	0	1	0	0	0	0	1 (3.4)
**Skin and subcutaneous tissue disorders**							
Ecchymosis	0	0	1	0	1	0	2 (6.9)
Eczema	1	0	0	0	0	0	1 (3.4)
Erythema	1	0	0	0	0	0	1 (3.4)
Night sweats	0	0	1	0	0	0	1 (3.4)
Petechiae	0	0	1	0	0	0	1 (3.4)
Pruritus	0	0	0	1	1	0	2 (6.9)
Rash	1	0	0	1	0	0	2 (6.9)
Rash macular	1	0	0	0	0	0	1 (3.4)
Rash maculo-papular	0	0	0	0	2	0	2 (6.9)
Skin mass	1	0	0	0	0	0	1 (3.4)
Skin ulcer	0	0	0	0	1	0	1 (3.4)
**Vascular disorders**							
Deep vein thrombosis	1	0	0	0	0	0	1 (3.4)
Hematoma	1	0	0	0	0	0	1 (3.4)
Hypertension	2	0	1	1	2	2	8 (27.6)
Hypotension	3	0	0	0	0	1	4 (13.8)
Orthostatic hypertension	0	0	0	1	0	0	1 (3.4)
Orthostatic hypotension	0	0	0	1	0	0	1 (3.4)
Systolic hypertension	0	0	0	0	1	0	1 (3.4)
Thrombophlebitis	1	0	0	0	0	0	1 (3.4)

Note: When the same event was reported twice for the same patient, it was only counted once, and the highest grade was captured.

Table S4. Incidence of adverse events CTCAE Grade 3-5 by MedDRA PT occurring in patients treated with OXA in Study OX1222 regardless of any relationship with the study drug OXi4503.

MedDRA SOC MedDRA PT	Cohorts						Total
	3.75 mg/m^2	4.68 mg/m^2	6.25 mg/m^2	7.81 mg/m^2	9.76 mg/m^2	12.2 mg/m^2	N = 29 n (%)
**Blood and lymphatic system disorders**							
**Anemia**	**4**	**2**	**1**	**1**	**2**	**1**	**11 (37.9)**
Grade 3	3	2	1	1	2	1	10 (34.5)
Grade 4	1	0	0	0	0	0	1 (3.4)
**Febrile neutropenia**	**3**	**3**	**4**	**3**	**3**	**3**	**19 (65.5)**
Grade 3	3	3	4	3	3	3	19 (65.5)
**Leukocytosis**	**1**	**0**	**0**	**0**	**0**	**0**	**1 (3.4)**
Grade 5	1	0	0	0	0	0	1 (3.4)
**Neutropenia**	**0**	**1**	**0**	**0**	**1**	**0**	**2 (6.9)**
Grade 4	0	1	0	0	1	0	2 (6.9)
**Pancytopenia**	**1**	**0**	**0**	**0**	**0**	**0**	**1 (3.4)**
Grade 4	1	0	0	0	0	0	1 (3.4)
**Thrombocytopenia**	**0**	**1**	**0**	**0**	**0**	**0**	**1 (3.4)**
Grade 4	0	1	0	0	0	0	1 (3.4)
**Cardiac disorders**							
**Tachycardia**	**1**	**0**	**0**	**0**	**1**	**0**	**2 (6.9)**
Grade 3	1	0	0	0	1	0	2 (6.9)
**Gastrointestinal disorders**							
**Rectal hemorrhage**	**0**	**0**	**1**	**0**	**0**	**0**	**1 (3.4)**
Grade 3	0	0	1	0	0	0	1 (3.4)
**Stomatitis**	**1**	**0**	**0**	**0**	**0**	**0**	**1 (3.4)**
Grade 3	1	0	0	0	0	0	1 (3.4)
**General disorders and administration site conditions**							
**Fatigue**	**1**	**0**	**0**	**0**	**0**	**0**	**1 (3.4)**
Grade 3	1	0	0	0	0	0	1 (3.4)
**Infections and infestations**							
**Atypical pneumonia**	**0**	**0**	**0**	**1**	**0**	**0**	**1 (3.4)**
Grade 3	0	0	0	1	0	0	1 (3.4)
**Bacteremia**	**0**	**0**	**1**	**1**	**0**	**0**	**2 (6.9)**
Grade 3	0	0	1	1	0	0	2 (6.9)
**Bacterial infection**	**0**	**1**	**0**	**0**	**0**	**0**	**1 (3.4)**
Grade 3	0	1	0	0	0	0	1 (3.4)
**Bronchopulmonary aspergillosis**	**0**	**0**	**1**	**0**	**0**	**0**	**1 (3.4)**
Grade 5	0	0	1	0	0	0	1 (3.4)
**Cellulitis**	**0**	**1**	**0**	**0**	**0**	**0**	**1 (3.4)**
Grade 3	0	1	0	0	0	0	1 (3.4)
**Liver abscess**	**1**	**0**	**0**	**0**	**0**	**0**	**1 (3.4)**

MedDRA SOC<br>MedDRA PT	Cohorts						Total
	3.75 mg/m^2	4.68 mg/m^2	6.25 mg/m^2	7.81 mg/m^2	9.76 mg/m^2	12.2 mg/m^2	N = 29<br>n (%)
Grade 3	1	0	0	0	0	0	1 (3.4)
**Periorbital cellulitis**	**1**	**0**	**0**	**0**	**0**	**0**	**1 (3.4)**
Grade 3	1	0	0	0	0	0	1 (3.4)
**Perirectal abscess**	**0**	**0**	**0**	**1**	**0**	**0**	**1 (3.4)**
Grade 3	0	0	0	1	0	0	1 (3.4)
**Pneumonia**	**0**	**0**	**0**	**0**	**0**	**2**	**2 (6.9)**
Grade 3	0	0	0	0	0	2	2 (6.9)
**Pneumonia klebsiella bacteremia**	**0**	**0**	**0**	**0**	**1**	**0**	**1 (3.4)**
Grade 3	0	0	0	0	1	0	1 (3.4)
**Sepsis**	**0**	**0**	**0**	**0**	**1**	**0**	**1 (3.4)**
Grade 4	0	0	0	0	1	0	1 (3.4)
**Sinusitis fungal**	**0**	**0**	**0**	**1**	**0**	**0**	**1 (3.4)**
Grade 5	0	0	0	1	0	0	1 (3.4)
**Streptococcal bacteremia**	**0**	**0**	**0**	**0**	**1**	**0**	**1 (3.4)**
Grade 3	0	0	0	0	1	0	1 (3.4)
Investigations							
**Alanine aminotransferase increased**	**0**	**0**	**0**	**0**	**1**	**1**	**2 (6.9)**
Grade 3	0	0	0	0	1	1	2 (6.9)
**Aspartate aminotransferase increased**	**1**	**0**	**0**	**0**	**0**	**1**	**2 (6.9)**
Grade 3	1	0	0	0	0	1	2 (6.9)
**Blood alkaline phosphatase increased**	**0**	**0**	**0**	**0**	**0**	**1**	**1 (3.4)**
Grade 3	0	0	0	0	0	1	1 (3.4)
**Blood bilirubin increased**	**0**	**0**	**0**	**0**	**0**	**1**	**1 (3.4)**
Grade 3	0	0	0	0	0	1	1 (3.4)
**Blood fibrinogen decreased**	**1**	**0**	**0**	**0**	**0**	**0**	**1 (3.4)**
Grade 3	1	0	0	0	0	0	1 (3.4)
**Ejection fraction decreased**	**0**	**0**	**0**	**1**	**0**	**0**	**1 (3.4)**
Grade 3	0	0	0	1	0	0	1 (3.4)
**Lipase increased**	**1**	**0**	**0**	**0**	**0**	**0**	**1 (3.4)**
Grade 3	1	0	0	0	0	0	1 (3.4)
**Neutrophil count decreased**	**2**	**0**	**0**	**1**	**1**	**0**	**4 (13.8)**
Grade 3	1	0	0	0	1	0	2 (6.9)
Grade 4	1	0	0	1	0	0	2 (6.9)
**Platelet count decreased**	**2**	**2**	**0**	**1**	**1**	**1**	**7 (24.1)**
Grade 3	0	0	0	0	1	0	1 (3.4)
Grade 4	2	2	0	1	0	1	6 (20.7)
**Prothrombin time prolonged**	**1**	**0**	**0**	**0**	**0**	**0**	**1 (3.4)**
Grade 3	1	0	0	0	0	0	1 (3.4)
**White blood cell count decreased**	**0**	**0**	**0**	**1**	**1**	**0**	**2 (6.9)**
Grade 4	0	0	0	1	1	0	1 (3.4)
**White blood cell count increased**	**1**	**0**	**0**	**0**	**0**	**0**	**1 (3.4)**

MedDRA SOC<br>MedDRA PT	Cohorts						Total
	3.75 mg/m^2	4.68 mg/m^2	6.25 mg/m^2	7.81 mg/m^2	9.76 mg/m^2	12.2 mg/m^2	N = 29<br>n (%)
Grade 3	1	0	0	0	0	0	1 (3.4)
**Metabolism and nutrition disorders**							
**Dehydration**	**1**	**0**	**0**	**0**	**0**	**0**	**1 (3.4)**
Grade 3	1	0	0	0	0	0	1 (3.4)
**Hyperglycemia**	**0**	**0**	**1**	**0**	**0**	**0**	**1 (3.4)**
Grade 3	0	0	1	0	0	0	1 (3.4)
**Hypermagnesemia**	**1**	**0**	**0**	**0**	**0**	**0**	**1 (3.4)**
Grade 3	1	0	0	0	0	0	1 (3.4)
**Hyperuricemia**	**1**	**0**	**0**	**0**	**0**	**0**	**1 (3.4)**
Grade 4	1	0	0	0	0	0	1 (3.4)
**Hypokalemia**	**2**	**1**	**0**	**0**	**0**	**0**	**3 (10.3)**
Grade 3	1	1	0	0	0	0	2 (6.9)
Grade 4	1	0	0	0	0	0	1 (3.4)
**Hyponatremia**	**0**	**0**	**0**	**1**	**0**	**0**	**1 (3.4)**
Grade 3	0	0	0	1	0	0	1 (3.4)
**Hypophosphatemia**	**1**	**0**	**1**	**0**	**0**	**0**	**2 (6.9)**
Grade 3	0	0	1	0	0	0	1 (3.4)
Grade 4	1	0	0	0	0	0	1 (3.4)
**Musculoskeletal and connective tissue disorders**							
**Back pain**	**1**	**0**	**0**	**0**	**0**	**0**	**1 (3.4)**
Grade 3	1	0	0	0	0	0	1 (3.4)
**Nervous system disorders**							
**Cognitive disorder**	**0**	**0**	**0**	**1**	**0**	**0**	**1 (3.4)**
Grade 3	0	0	0	1	0	0	1 (3.4)
**Dysarthria**	**0**	**0**	**0**	**0**	**0**	**1**	**1 (3.4)**
Grade 3	0	0	0	0	0	1	1 (3.4)
**Embolic stroke**	**0**	**0**	**0**	**1**	**0**	**0**	**1 (3.4)**
Grade 4	0	0	0	1	0	0	1 (3.4)
**Facial paresis**	**0**	**0**	**0**	**1**	**0**	**0**	**1 (3.4)**
Grade 3	0	0	0	1	0	0	1 (3.4)
**Renal and urinary disorders**							
**Acute kidney injury**	**0**	**1**	**0**	**0**	**0**	**0**	**1 (3.4)**
Grade 4	0	1	0	0	0	0	1 (3.4)
**Respiratory, thoracic and mediastinal disorders**							
**Hypoxia**	**0**	**0**	**1**	**0**	**1**	**1**	**3 (10.3)**
Grade 3	0	0	1	0	1	1	3 (10.3)
**Pulmonary edema**	**0**	**0**	**0**	**0**	**0**	**1**	**1 (3.4)**
Grade 3	0	0	0	0	0	1	1 (3.4)
**Respiratory failure**	**0**	**0**	**0**	**0**	**0**	**1**	**1 (3.4)**
Grade 4	0	0	0	0	0	1	1 (3.4)
**Vascular disorders**							
**Hypertension**	**1**	**0**	**0**	**1**	**1**	**2**	**5 (17.2)**

MedDRA SOC<br>MedDRA PT	Cohorts						Total
	3.75 mg/m^2	4.68 mg/m^2	6.25 mg/m^2	7.81 mg/m^2	9.76 mg/m^2	12.2 mg/m^2	N = 29<br>n (%)
Grade 3	1	0	0	1	1	2	5 (17.2)
**Hypotension**	**1**	**0**	**0**	**0**	**0**	**1**	**2 (6.9)**
Grade 3	0	0	0	0	0	1	1 (3.4)
Grade 4	1	0	0	0	0	0	1 (3.4)
**Orthostatic hypotension**	**0**	**0**	**0**	**1**	**0**	**0**	**1 (3.4)**
Grade 3	0	0	0	1	0	0	1 (3.4)

Note: When the same event was reported twice for the same patient, it was only counted once, and the highest grade was captured.

Table S5. All Severe Adverse Events (SAEs) Occurring After Treatment with OXA in the OX1222 study.

Patient No.	Cohort#	SAE Reported Term (CTCAE Grade)	Relatedness with OXi4503	SAE Outcome	Action Taken
101-002	1	Leukocytosis [Disease Progression] (5)	Not related	Not recovered/Fatal	Permanently discontinued
103-002	1	Tachycardia (3)	Not related	Recovered, without sequelae	None
103-002	1	Pancytopenia (4)	Not related	Recovered, without sequelae	None
103-002	1	Oral Mucositis (3)	Not related	Recovered, without sequelae	None
103-003	1	Neutropenic Fever (3)	Not related	Recovered, without sequelae	None
103-003	1	Hepatic Abscess (3)	Not related	Recovered, with sequelae	None
106-001	1	Periorbital cellulitis (3)	Not related	Recovered, with sequelae	None
106-001	1	Hypotension (4)	Not related	Recovered, without sequelae	None
106-004	1	Neutropenic Fever (3)	Probably Not Related	Recovered, without sequelae	None
106-004	1	Pyrexia (1)	Possibly related	Recovered, without sequelae	None
103-005	2	Neutropenic fever (3)	Not related	Recovered, without sequelae	None
103-005	2	Gram negative rod, bacterial infection (3)	Probably Not Related	Recovered, without sequelae	None
106-005	2	Cellulitis of lower left leg [shin] (3)	Not related	Recovered, without sequelae	None
106-005	2	Febrile Neutropenia (3)	Not related	Recovered, without sequelae	None
106-006	2	Neutropenic Fever (3)	Possibly related	Recovered, without sequelae	None
106-006	2	Pyrexia (2)	Possibly related	Not recovered	None
106-006	2	Worsening of Acute Kidney Injury (4)	Not related	Recovered, without sequelae	Dose delayed for OXi4503 and ARA-C
103-009	3	Febrile Neutropenia (3)	Not related	Recovered, without sequelae	None
103-009	3	Rectal Bleeding (3)	Probably Not Related	Recovered, without sequelae	None
101-005	3	Bacteremia (3)	Probably Not Related	Recovered, without sequelae	None
107-002	3	Aspergillus pneumonia (5)	Not related	Not recovered/Fatal	Permanently discontinued
103-010	4	Neutropenic fever (3)	Related	Recovered, without sequelae	None
103-010	4	Perirectal Abscess (3)	Not related	Recovered, without sequelae	None
106-008	4	Orthostatic Hypotension (3)	Not related	Recovered, without sequelae	None
106-008	4	Invasive Fungal Sinusitis (5)	Not related	Not recovered/Fatal	None
106-008	4	Stroke (embolic) (4)	Probably Not Related	Recovered, with sequelae	Permanently discontinued

Patient No.	Cohort#	SAE Reported Term (CTCAE Grade)	Relatedness with OXi4503	SAE Outcome	Action Taken
103-011	5	Klebsiella pneumonia bacteremia (3)	Not related	Recovered, without sequelae	Dose delayed for ARA-C
106-009	5	Febrile Neutropenia (3)	Probably Not Related	Recovered, without sequelae	None
107-003	5	Neutropenia (4)	Unlikely Related	Recovered, without sequelae	None
107-008	5	Sepsis (4)	Unrelated	Recovered, without sequelae	None
107-008	5	Progressive disease (5)	Unrelated	Not recovered/Fatal	None
103-012	6	Acute hypoxic respiratory failure (4) secondary to pneumonia	Possibly related	Not recovered	None
106-011	6	Hypotension (3)	Possibly related	Recovered	None
106-011	6	Lung Infection (multi-focal pneumonia) (3)	Not related	Not recovered	None

Table S6. Incidence of SAEs occurring after treatment with OXA by MedDRA PT (any CTCAE Grade) regardless of any relationship with the study drug OXi4503.

MedDRA SOC MedDRA PT	Cohorts						Total
	3.75 mg/m²	4.68 mg/m²	6.25 mg/m²	7.81 mg/m²	9.76 mg/m²	12.2 mg/m²	N = 29 n (%)
**Blood and lymphatic system disorders**							
**Febrile neutropenia**	**2**	**3**	**1**	**1**	**1**	**0**	**8 (27.6)**
Grade 3	2	3	1	1	1	0	8 (27.6)
**Leukocytosis**	**1**	**0**	**0**	**0**	**0**	**0**	**1 (3.4)**
Grade 5	1	0	0	0	0	0	1 (3.4)
**Neutropenia**	**0**	**0**	**0**	**0**	**1**	**0**	**1 (3.4)**
Grade 4	0	0	0	0	1	0	1 (3.4)
**Pancytopenia**	**1**	**0**	**0**	**0**	**0**	**0**	**1 (3.4)**
Grade 4	1	0	0	0	0	0	1 (3.4)
**Cardiac disorders**							
**Tachycardia**	**1**	**0**	**0**	**0**	**0**	**0**	**1 (3.4)**
Grade 3	1	0	0	0	0	0	1 (3.4)
**Gastrointestinal disorders**							
**Rectal hemorrhage**	**0**	**0**	**1**	**0**	**0**	**0**	**1 (3.4)**
Grade 3	0	0	1	0	0	0	1 (3.4)
**Stomatitis**	**1**	**0**	**0**	**0**	**0**	**0**	**1 (3.4)**
Grade 3	1	0	0	0	0	0	1 (3.4)
General Disorders Pyrexia (Grade ½)	1	1	0	0	0	0	2 (6.9)
**Infections and infestations**							
**Bacteremia**	**0**	**0**	**1**	**0**	**0**	**0**	**1 (3.4)**
Grade 3	0	0	1	0	0	0	1 (3.4)
**Bacterial infection**	**0**	**1**	**0**	**0**	**0**	**0**	**1 (3.4)**
Grade 3	0	1	0	0	0	0	1 (3.4)
**Bronchopulmonary aspergillosis**	**0**	**0**	**1**	**0**	**0**	**0**	**1 (3.4)**
Grade 5	0	0	1	0	0	0	1 (3.4)
**Cellulitis**	**0**	**1**	**0**	**0**	**0**	**0**	**1 (3.4)**
Grade 3	0	1	0	0	0	0	1 (3.4)
**Liver abscess**	**1**	**0**	**0**	**0**	**0**	**0**	**1 (3.4)**
Grade 3	1	0	0	0	0	0	1 (3.4)
**Periorbital cellulitis**	**1**	**0**	**0**	**0**	**0**	**0**	**1 (3.4)**
Grade 3	1	0	0	0	0	0	1 (3.4)
**Perirectal abscess**	**0**	**0**	**0**	**1**	**0**	**0**	**1 (3.4)**
Grade 3	0	0	0	1	0	0	1 (3.4)
**Pneumonia**	**0**	**0**	**0**	**0**	**0**	**1**	**1 (3.4)**
Grade 3	0	0	0	0	0	1	1 (3.4)
**Pneumonia klebsiella bacteremia**	**0**	**0**	**0**	**0**	**1**	**0**	**1 (3.4)**
Grade 3	0	0	0	0	1	0	1 (3.4)
**Sepsis**	0	0	0	0	1	0	**1 (3.4)**

MedDRA SOC MedDRA PT	Cohorts						Total
	3.75 mg/m²	4.68 mg/m²	6.25 mg/m²	7.81 mg/m²	9.76 mg/m²	12.2 mg/m²	N = 29 n (%)
Grade 4	0	0	0	0	1	0	1 (3.4)
**Sinusitis fungal**	**0**	**0**	**0**	**1**	**0**	**0**	**1 (3.4)**
Grade 5	0	0	0	1	0	0	1 (3.4)
**Nervous system disorders**							
**Embolic stroke**	**0**	**0**	**0**	**1**	**0**	**0**	**1 (3.4)**
Grade 4	0	0	0	1	0	0	1 (3.4)
**Renal and urinary disorders**							
**Acute kidney injury**	**0**	**1**	**0**	**0**	**0**	**0**	**1(3.4)**
Grade 4	0	1	0	0	0	0	1 (3.4)
**Respiratory, thoracic and mediastinal disorders**							
**Respiratory failure**	**0**	**0**	**0**	**0**	**0**	**1**	**1 (3.4)**
Grade 4	0	0	0	0	0	1	1 (3.4)
**Vascular disorders**							
**Hypotension**	**1**	**0**	**0**	**0**	**0**	**1**	**2 (6.9)**
Grade 3	0	0	0	0	0	1	1 (3.4)
Grade 4	1	0	0	0	0	0	1 (3.4)
**Orthostatic hypotension**	**0**	**0**	**0**	**1**	**0**	**0**	**1 (3.4)**
Grade 3	0	0	0	1	0	0	1 (3.4)

Note: When the same event was reported twice for the same patient, it was only counted once, and the highest grade was captured.

Table S7. Incidence of laboratory abnormalities* CTCAE Grade 3-4 by MedDRA PT regardless of any relationship with the study drug OXi4503.

MedDRA SOC MedDRA PT	CTCAE Grade	Cohorts						Total
		3.75 mg/m²	4.68 mg/m²	6.25 mg/m²	7.81 mg/m²	9.76 mg/m²	12.2 mg/m²	N = 29 n (%)
**Investigations**								
Alanine aminotransferase increased	Grade 3	0	0	0	0	1	1	2 (6.9)
Aspartate aminotransferase increased	Grade 3	1	0	0	0	0	1	2 (6.9)
Blood alkaline phosphatase increased	Grade 3	0	0	0	0	0	1	1 (3.4)
Blood bilirubin increased	Grade 3	0	0	0	0	0	1	1 (3.4)
Blood fibrinogen decreased	Grade 3	1	0	0	0	0	0	1 (3.4)
Lipase increased	Grade 3	1	0	0	0	0	0	1 (3.4)
Neutrophil count decreased	Grade 3	1	0	0	0	2	0	2 (6.9)
	Grade 4	1	0	0	1	0	0	2 (6.9)
Platelet count decreased	Grade 3	0	0	0	0	1	0	1 (3.4)
	Grade 4	2	2	0	1	0	1	6 (20.7)
Prothrombin time prolonged	Grade 3	1	0	0	0	0	0	1(3.4)
White blood cell count decreased	Grade 4	0	0	0	1	1	0	2 (6.9)
White blood cell count increased	Grade 3	1	0	0	0	0	0	1 (3.4)

Note: When the same event was reported twice for the same patient, it was only counted once, and the highest grade was captured. *This table includes lab abnormalities reported as adverse events.

Table S8. Incidence of OXi4503-related AEs by MedDRA PT (any CTCAE Grade).

MedDRA SOC<br>MedDRA PT	Cohorts						Total
	3.75 mg/m²	4.68 mg/m²	6.25 mg/m²	7.81 mg/m²	9.76 mg/m²	12.2 mg/m²	N = 29 n (%)
**Blood and lymphatic system disorders**							
**Anemia**	**1**	**1**	**0**	**1**	**1**	**0**	**4 (13.8)**
Definitely related	1	0	0	0	0	0	1 (3.4)
Possibly related	0	1	0	1	1	0	3 (10.3)
Probably related	1	0	0	0	0	0	1 (3.4)
**Disseminated intravascular coagulation**	**0**	**0**	**0**	**0**	**0**	**1**	**1 (3.4)**
Definitely related	0	0	0	0	0	1	1 (3.4)
**Febrile neutropenia**	**0**	**1**	**2**	**3**	**1**	**1**	**8 (27.6)**
Definitely related	0	0	0	1	0	0	1 (3.4)
Possibly related	0	1	0	1	1	1	4 (13.8)
Probably related	0	0	2	1	0	0	3 (10.3)
**Neutropenia**	**0**	**1**	**0**	**0**	**0**	**0**	**1 (3.4)**
Possibly related	0	1	0	0	0	0	1 (3.4)
**Pancytopenia**	**0**	**0**	**1**	**0**	**0**	**0**	**1 (3.4)**
Possibly related	0	0	1	0	0	0	1 (3.4)
**Thrombocytopenia**	**0**	**1**	**0**	**0**	**0**	**0**	**1 (3.4)**
Possibly related	0	1	0	0	0	0	1 (3.4)
**Gastrointestinal disorders**							
**Constipation**	**1**	**0**	**0**	**0**	**0**	**0**	**1 (3.4)**
Possibly related	1	0	0	0	0	0	1 (3.4)
**Diarrhea**	**0**	**0**	**1**	**0**	**0**	**0**	**1 (3.4)**
Possibly related	0	0	1	0	0	0	1 (3.4)
**Nausea**	**1**	**0**	**1**	**0**	**2**	**0**	**4 (13.8)**
Possibly related	1	0	0	0	2	0	3 (10.3)
Probably related	0	0	1	0	0	0	1 (3.4)
**Proctalgia**	**0**	**0**	**1**	**0**	**0**	**0**	**1 (3.4)**
Probably related	0	0	1	0	0	0	1 (3.4)
**Vomiting**	**1**	**0**	**0**	**0**	**1**	**0**	**2 (6.9)**
Possibly related	0	0	0	0	1	0	1 (3.4)
Probably related	0	0	1	0	0	0	1 (3.4)
**General disorders and administration site conditions**							
**Chills**	**0**	**0**	**3**	**0**	**0**	**0**	**3 (10.3)**
Probably related	0	0	1	0	0	0	1 (3.4)
Possibly related	0	0	2	0	0	0	2 (6.9)
**Edema**	**1**	**0**	**0**	**0**	**0**	**0**	**1 (3.4)**
Possibly related	1	0	0	0	0	0	1 (3.4)
**Pain**	**0**	**0**	**0**	**1**	**0**	**0**	**1 (3.4)**
Possibly related	0	0	0	1	0	0	1 (3.4)
**Pyrexia**	**0**	**0**	**2**	**0**	**0**	**0**	**2 (6.9)**
Probably related	0	0	2	0	0	0	2 (6.9)
**Infections and infestations**							

MedDRA SOC MedDRA PT	Cohorts						Total
	3.75 mg/m^2	4.68 mg/m^2	6.25 mg/m^2	7.81 mg/m^2	9.76 mg/m^2	12.2 mg/m^2	N = 29 n (%)
**Bacteremia**	**0**	**0**	**1**	**0**	**0**	**0**	**1 (3.4)**
Probably related	0	0	0	1	0	0	1 (3.4)
**Pneumonia**	**0**	**0**	**0**	**0**	**0**	**1**	**1 (3.4)**
Possibly related	0	0	0	0	0	1	1 (3.4)
**Investigations**							
**Activated partial thromboplastin time prolonged**	**1**	**0**	**0**	**0**	**0**	**0**	**1 (3.4)**
Probably related	1	0	0	0	0	0	1 (3.4)
**Alanine aminotransferase increased**	**1**	**0**	**0**	**0**	**0**	**1**	**2 (6.9)**
Definitely related	1	0	0	0	0	0	**1 (3.4)**
Possibly related	0	0	0	0	0	1	1 (3.4)
**Aspartate aminotransferase increased**	**1**	**0**	**0**	**0**	**0**	**1**	**2 (6.9)**
Definitely related	1	0	0	0	0	0	**1 (3.4)**
Possibly related	0	0	0	0	0	1	1 (3.4)
**Blood bilirubin increased**	**0**	**0**	**0**	**1**	**0**	**1**	**2 (6.9)**
Possibly related	0	0	0	1	0	1	2 (6.9)
**Blood fibrinogen decreased**	**2**	**0**	**0**	**1**	**1**	**1**	**5 (17.2)**
Possibly related	0	0	0	1	0	0	1 (3.4)
Definitely related	2	0	0	0	1	1	4 (13.8)
**Fibrin D-dimer increased**	**0**	**0**	**0**	**0**	**1**	**0**	**1 (3.4)**
Probably related	0	0	0	0	1	0	1 (3.4)
**International normalized ratio increased**	**0**	**0**	**0**	**1**	**0**	**0**	**1 (3.4)**
Possibly related	0	0	0	1	0	0	1 (3.4)
**Neutrophil count decreased**	**2**	**0**	**0**	**1**	**1**	**0**	**4 (13.8)**
Possibly related	1	0	0	0	1	0	2 (6.9)
Possibly related	1	0	0	1	0	0	2 (6.9)
**Platelet count decreased**	**2**	**1**	**0**	**1**	**1**	**0**	**5 (17.2)**
Possibly related	0	1	0	1	1	0	3 (10.3)
Probably related	2	0	0	0	0	0	2 (6.9)
**Prothrombin time prolonged**	**1**	**0**	**0**	**0**	**0**	**0**	**1 (3.4)**
Probably related	1	0	0	0	0	0	1 (3.4)
**White blood cell count decreased**	**0**	**0**	**0**	**1**	**1**	**0**	**2 (6.9)**
Possibly related	0	0	0	1	1	0	2 (6.9)
**Metabolism and nutrition disorders**							
**Hyperammonemia**	**0**	**0**	**0**	**0**	**0**	**1**	**1 (3.4)**
Possibly related	0	0	0	0	0	1	1 (3.4)
**Hyperkalemia**	**0**	**1**	**0**	**0**	**0**	**0**	**1 (3.4)**
Possibly related	0	1	0	0	0	0	1 (3.4)
**Hyperuricemia**	**0**	**0**	**0**	**1**	**0**	**0**	**1 (3.4)**
Possibly related	0	0	0	1	0	0	1 (3.4)
**Hypocalcemia**	**0**	**1**	**0**	**0**	**0**	**0**	**1 (3.4)**
Possibly related	0	1	0	0	0	0	1 (3.4)

MedDRA SOC<br>MedDRA PT	Cohorts						Total
	3.75 mg/m²	4.68 mg/m²	6.25 mg/m²	7.81 mg/m²	9.76 mg/m²	12.2 mg/m²	N = 29<br>n (%)
**Hypokalemia**	**1**	**0**	**0**	**0**	**0**	**0**	**1 (3.4)**
Possibly related	1	0	0	0	0	0	1 (3.4)
**Hypomagnesemia**	**0**	**0**	**0**	**0**	**0**	**1**	**1 (3.4)**
Possibly related	0	0	0	0	0	1	1 (3.4)
**Musculoskeletal and connective tissue disorders**							
**Back pain**	**0**	**0**	**0**	**1**	**0**	**0**	**1 (3.4)**
Possibly related	0	0	0	1	0	0	1 (3.4)
**Bone pain**	**0**	**0**	**1**	**1**	**1**	**1**	**4 (13.8)**
Definitely related	0	0	0	0	1	1	2 (6.9)
Possibly related	0	0	1	1	0	0	2 (6.9)
**Pain in jaw**	**0**	**0**	**0**	**0**	**1**	**0**	**1 (3.4)**
Definitely related	0	0	0	0	1	0	1 (3.4)
**Nervous system disorders**							
**Headache**	**0**	**0**	**0**	**0**	**1**	**1**	**2 (6.9)**
Definitely related	0	0	0	0	0	1	1 (3.4)
Possibly related	0	0	0	0	1	0	1 (3.4)
**Psychiatric disorders**							
**Delirium**	**0**	**0**	**0**	**0**	**0**	**1**	**1 (3.4)**
Possibly related	0	0	0	0	0	1	1 (3.4)
**Renal and urinary disorders**							
**Urinary incontinence**	**0**	**0**	**0**	**0**	**0**	**1**	**1 (3.4)**
Definitely related	0	0	0	0	0	1	1 (3.4)
**Respiratory, thoracic and mediastinal disorders**							
**Respiratory failure**	**0**	**0**	**0**	**0**	**0**	**1**	**1 (3.4)**
Possibly related	0	0	0	0	0	1	1 (3.4)
**Skin and subcutaneous tissue disorders**							
**Petechiae**	**0**	**0**	**1**	**0**	**0**	**0**	**1 (3.4)**
Possibly related	0	0	1	0	0	0	1 (3.4)
**Vascular disorders**							
**Hypertension**	**1**	**0**	**1**	**1**	**2**	**2**	**7 (24.1)**
Possibly related	1	0	1	0	0	1	2 (6.9)
Probably related	0	0	0	1	2	0	3 (10.3)
Definitely related	0	0	0	0	1	1	2 (6.9)
**Hypotension**	**0**	**0**	**0**	**0**	**0**	**1**	**1 (3.4)**
Possibly related	0	0	0	0	0	1	1 (3.4)

Note: When the same event was reported twice for the same patient, it was only counted once, and the highest grade was captured.

Table S9. OXi4503-related Grade 3/4 AEs and Survival Outcome in Study OX1222 patients treated with OXA. There were no incidences of drug-related Grade 5 adverse events.

Patient ID	Cohort#	Reported Term (Grade)	Related SAE (Yes/No)	EOS Reason	Other Therapy post EOS	Time to Death or Last FU after C1D1 (Days)	Survival Status at Last FU	Cause of Death
101-002	1	Fibrinogen decrease (3)<br>Hypokalemia (3)<br>Neutrophil count decrease (3)	No<br>No<br>No	LOE	HU	30	D	PD
106-004	1	Hypertension (3)	No	CRi→DLI	DLI	535	D	PD
103-004	1	Platelet count decrease (4)<br>Neutrophil count decrease (4)	No<br>No	LOE	Not reported	106	D	PD
103-002	1	PT increased (3)<br>AST increase (3)<br>Anemia(4),<br>Platelet count decreased (4)	No<br>No<br>No<br>No	LOE	Not reported	77	D	PD
103-003	1	None	No	PD	Not reported	87	D	PD
106-001	1	None	No	PD	Not reported	172	D	PD
106-003	1	None	No	NE	Not reported	40	D	NR/LFU
106-005	2	None	No	PD	7+3;HU	285	D	PD
106-006	2	Febrile neutropenia (3 - SAE)<br>Worsened anemia (3)<br>Platelet decrease (4),<br>Neutropenia (4)	Yes<br>No<br>No<br>No	CRi	Mylotarg, 5-AC	521	D	PD
103-005	2	Platelet count decrease (4)	No	LOE	CAFFdA, Allo-PBSCT	212	D	PD
103-008	2	None	No	LOE	None	48	D	PD
101-005	3	Febrile neutropenia (3)	No	LOE	Jakafi	119	D	PD
103-009	3	None	No	CR→SCT	SCT, 5AZA	434	D	PD
107-001	3	Febrile neutropenia (3)	Yes	LOE	IP	191	D	NR
107-002	3	None	No	LOE	NA	59	D	Aspergillus pneumonia (Unrelated)

Patient ID	Cohort#	Reported Term (Grade)	Related SAE (Yes/No)	EOS Reason	Other Therapy post EOS	Time to Death or Last FU after C1D1 (Days)	Survival Status at Last FU	Cause of Death
106-008	4	Febrile neutropenia(3),<br>WBC decrease (4),<br>Hypertension (3)	No<br>No<br>No	Unrelated SAE	NA	61	D	Invasive fungal sinusitis (Unrelated)
103-010	4	Febrile neutropenia (3 - SAE)<br>Platelet count decrease (4),<br>Anemia worsening (3)	Yes<br>No<br>No	LOE	5-AC, Nexavar	135	D	PD
101-006	4	Febrile neutropenia (3)<br>Bacteremia (3)<br>Neutrophil count decreased (4)	No<br>No<br>No	LOE	Velcade, HU	113	D	PD
106-009	5	Neutrophil count decrease (3),<br>Platelet count decrease(3),<br>WBC decrease (4),<br>Hypertension (3)	No<br>No<br>No<br>No	LOE	5AZA	232	D	NR/HOS
107-003	5	Febrile neutropenia (3)	No	LOE	Allo PBSCT	720	A	NA
103-011	5	Worsening anemia (3)	No	LOE	LDAC	298	D	PD
101-007	5	None	No	LOE	NR	393	D	PD
107-004	5	None	No	LOE	NA	114	D	PD
107-007	5	None	No	LOE	NA	69	D	PD
107-008	5	None	No	LOE	NA	66	D	PD
103-012	6	Hypertension (3),<br>AST increase (3),<br>Febrile neutropenia (3),<br>Pneumonia (3),<br>Acute hypoxic respiratory failure resulting from pneumonia (4 – SAE)	No<br>No<br>No<br>No<br>Yes	PD	NA	34	D	PD and SAE
103-013	6	None	No	LOE	-	77	D	PD
106-010	6	Hypertension (3)	No	LOE and ARA-C NT	NR/LFU	127	D	NR/LFU
106-011	6	Hypotension (3)<br>Blood bilirubin increased (3)	Yes<br>No	LOE	NR/LFU	58	D	PD

C1D1: Cycle 1 Day 1; FU: Follow-up; AML: acute myelogenous leukemia; MDS: Myelodysplastic Syndromes; PD: Progressive disease; CR i: Complete response with incomplete hematologic recovery; SD/RES: Stable disease/Refractory; NE: Not evaluable; CR: Complete response; A: Alive; D: Dead; EOS: End of Study; NA: Not available; LOE: Loss of efficacy; SAE: serious adverse event; HU: hydroxyurea; DLI: donor leukocyte infusion; 5-AC: 5-Azacytidine; PBSCT: Peripheral blood stem cell transplantation; SCT: stem cell transplant; 5AZA: deoxyazacytidine . NT: neurotoxicity.

Table S10. All study drug-related Grade 3–4 adverse events* reported in Study OX1222. There were no incidences of drug-related Grade 5 adverse events.

Patient No.	Cohort#	AE Reported Term (CTCAE Grade)	Relatedness with OXi4503	Outcome	Action Taken with OXi4503
101-002	1	Hypokalemia (3)	Possibly related	Recovered, without sequelae	None
		Blood fibrinogen decreased (3)	Definitely related	Recovered, without sequelae	None
		Neutrophil count decreased (3)	Possibly related	Recovered, without sequelae	None
103-002	1	Prothrombin time prolonged (3)	Probably related	Recovered, without sequelae	None
		Anemia (3)	Definitely related	Recovered, without sequelae	None
		Anemia (4)	Probably related	Recovered, without sequelae	None
		Aspartate aminotransferase increased (3)	Definitely related	Recovered, without sequelae	None
		Platelet count decreased (4)	Probably related	Recovered, without sequelae	None
103-004	1	Platelet count decreased (4)	Probably related	Recovered, without sequelae	None
		Neutrophil count decreased (4)	Possibly related	Recovered, without sequelae	None
106-004	1	Hypertension (3)	Possibly related	Recovered, without sequelae	None
106-006	2	Febrile neutropenia (3)	Possibly related	Recovered, without sequelae	None
		Anemia, worsening (3)	Possibly related	Recovered, without sequelae	None
		Thrombocytopenia (4)	Possibly related	Recovered, without sequelae	None
		Neutropenia (3)	Possibly related	Recovered, without sequelae	None
		Neutropenia (4)	Possibly related	Recovered, without sequelae	None
103-005	2	Platelet count decreased (4)	Possibly related	Recovered, without sequelae	None
101-005	3	Febrile neutropenia (3)	Probably related	Recovered, without sequelae	None
107-001	3	Febrile neutropenia (3)	Probably related	Recovered, without sequelae	None
103-010	4	Febrile neutropenia (3)	Definitely related	Recovered, without sequelae	None
		Anemia, worsening (3)	Possibly related	Recovering	None
		Platelet count decreased (4)	Possibly related	Recovering	None

Patient No.	Cohort#	AE Reported Term (CTCAE Grade)	Relatedness with OXi4503	Outcome	Action Taken with OXi4503
106-008	4	Febrile neutropenia (3)	Possibly related	Recovered, without sequelae	None
		White blood cell count decreased (4)	Possibly related	Not recovered	None
		White blood cell count decreased (3)	Possibly related	Not recovered	None
		Hypertension (3)	Probably related	Recovered, without sequelae	None
101-006	4	Febrile neutropenia (3)	Probably related	Recovered, without sequelae	None
		Bacteremia (3)	Probably related	Recovered, without sequelae	None
		Neutrophil count decreased (3)	Probably related	Recovered, without sequelae	None
		Neutrophil count decreased (4)	Possibly related	Recovered, without sequelae	None
103-011	5	Anemia, worsening (3)	Possibly related	Recovered, without sequelae	None
106-009	5	Neutrophil count decreased (3)	Possibly related	Not recovered	None
		Platelet count decreased (3)	Possibly related	Not recovered	None
		White blood cell count decreased (4)	Possibly related	Not recovered	None
		Hypertension (3)	Definitely related	Recovered, without sequelae	None
107-003	5	Febrile neutropenia (3)	Possibly related	Recovered, without sequelae	None
103-012	6	Hypertension (3)	Definitely related	Recovered, without sequelae	None
		Aspartate aminotransferase increased (3)	Possibly related	Recovered, without sequelae	None
		Febrile neutropenia (3)	Possibly related	Not recovered	None
		Pneumonia (3)	Possibly related	Not recovered	None
		Acute Hypoxic Respiratory failure (4)	Possibly related	Not Recovered	None
		Hypertension (3)	Definitely related	Recovered, without sequelae	None
106-010	6	Hypertension (3)	Probably related	Recovering	None
106-011	6	Hypotension (3)	Possibly related	Recovered	None
		Blood bilirubin increased (3)	Possibly related	Recovering	None

*Multiple events for the same term and patient have been reported as 1 event only unless same event was reported for 2 different Grades i.e., worsened.

© 2019 by the authors. Licensee MDPI, Basel, Switzerland. This article is an open access article distributed under the terms and conditions of the Creative Commons Attribution (CC BY) license (http://creativecommons.org/licenses/by/4.0/).
